# Floral miniaturisation and autogamy in boreal-arctic plants are epitomised by Iceland’s most frequent orchid, *Platanthera hyperborea*

**DOI:** 10.7717/peerj.894

**Published:** 2015-04-14

**Authors:** Richard M. Bateman, Gábor Sramkó, Paula J. Rudall

**Affiliations:** 1 Royal Botanic Gardens Kew, Richmond, United Kingdom; 2 Department of Botany, University of Debrecen, Debrecen, Hungary

**Keywords:** Allometry, Autogamy, Baker’s rule, Functional constraint, Iceland, Endemism, Internal Transcribed Spacer, Island biogeography, Migration, Molecular phylogeny, Orchid, Morphometrics, Paedomorphosis, *Platanthera hyperborea*, Speciation

## Abstract

**Background and Aims**. This paper concludes our series of publications comparing island and mainland speciation in European butterfly-orchids, by studying the morphology, phylogenetics and reproductive biology of the controversial circum-arctic species *Platanthera* (*Limnorchis*) *hyperborea*—the most frequent of seven Icelandic orchids. We draw particular attention to its phylogenetic placement, remarkable reproductive biology and morphological convergence on other *Platanthera* lineages through floral miniaturisation.

**Methods**. Five populations of *P. hyperborea* in southwest Iceland were measured for 33 morphological characters and subjected to detailed multivariate and univariate analyses, supported by light and scanning electron microscopy of selected flowers. Representative samples from six populations were sequenced for nrITS and placed in a taxonomically broader phylogenetic matrix derived from previous studies.

**Key Results** *.* Section *Limnorchis* consists of three distinct ITS-delimited clades based on *P. stricta*, *P. sparsifolia–limosa–aquilonis* and *P. dilatata–hyperborea*. Within the latter group, supposed species boundaries overlap; instead, the data indicate a crude stepwise series of ribotypic transitions extending eastward from North America to Iceland. Morphometric data failed to identify any taxonomically meaningful partitions among Icelandic *P. hyperborea* populations, despite the presence of a distinct and apparently plesiomorphic ribotype at the most glacially influenced habitat sampled. Microscopic study of the flowers revealed several distinguishing features (some not previously reported), including resupinate lateral sepals, toothed bract margins, club-shaped papillae shared by both the interior of the labellar spur and the stigmatic surface, and an exceptionally adhesive stigma that is reliably covered in disaggregated pollen masses prior to anthesis; auricles are absent.

**Conclusions.** Ribotypes suggest that Icelandic *P. hyperborea* represents the terminus of a migration route that may have begun in East Asia before passing through North America and presumably Greenland. The incohesive pollinia, rapidly desiccating anther locules, weakly developed rostellum, exceptionally adhesive stigma and the close juxtaposition of compact male and female reproductive organs together conspire to cause routine autogamy and frequent cleistogamy, despite the continued production of substantial nectar reservoirs in the spur and consequent ongoing attraction to the flowers of insects, including mosquitoes. When considered in combination with independently derived lineages of *Platanthera* on the Azorean and Hawaiian archipelagos also bearing small green flowers, our observations show allometric and paedomorphic reductions in flower size as the primary evolutionary driver, but also indicate strong developmental and functional constraints.

## Introduction

This paper is the fourth and final element of an integrated monograph of European members of the genus *Platanthera.* The three previous studies considered only species that proved through molecular analyses to be members of *Platanthera* section *Platanthera* (Bateman, James & Rudall, 2012; Bateman, Rudall & Moura, 2013; Bateman et al., 2014). However, two further species of *Platanthera* occur in Europe, both specialising in boreal environments. *Platanthera oligantha* Turcz. occurs in northern Scandinavia and Arctic Russia; its morphological assignment to Section *Lysiella* was recently confirmed using DNA evidence (Bateman et al., 2009). In contrast, *P. hyperborea* (L.) Lindl. (Lindley, 1835), is not found in mainland Europe but rather within ‘Greater Europe’ it is confined to Iceland, from where the species was first described in 1767 (Linné, 1767). Traditional morphological studies have placed this species in the dominantly North American–northeast Asian Section *Limnorchis* (Rydberg, 1900).

Species circumscription within Section *Limnorchis* has long been controversial. At the turn of the 20th Century, Kränzlin (1897–1904) chose to recognise only one highly polymorphic species, whereas the radical alternative advocated by Rydberg (1900) required not only 24 species but also their segregation as a full genus, *Limnorchis* Rydb. Indeed, putative species in the group are still being described; recent examples include *P. tescamnis* in the vicinity of the southern Rocky Mountains (Sheviak & Jennings, 2006) and *P. yosemitensis*, a supposed endemic apparently consisting of a single metapopulation in the Sierra Nevada Mountains of California (Colwell, Sheviak & Moore, 2007). Sadly, these putative species are still being established entirely on the basis of traditional taxonomic approaches (i.e., in the absence of morphometric, karyotypic and genetic data) and in a piecemeal fashion (i.e., in the absence of rigorous group-wide comparison of the required multiple datasets). Consequently, as noted by Luer (1975, p. 223) when addressing this subtly variable and enigmatic group, “attempts at identification are often arbitrary.”

**Figure 1:**
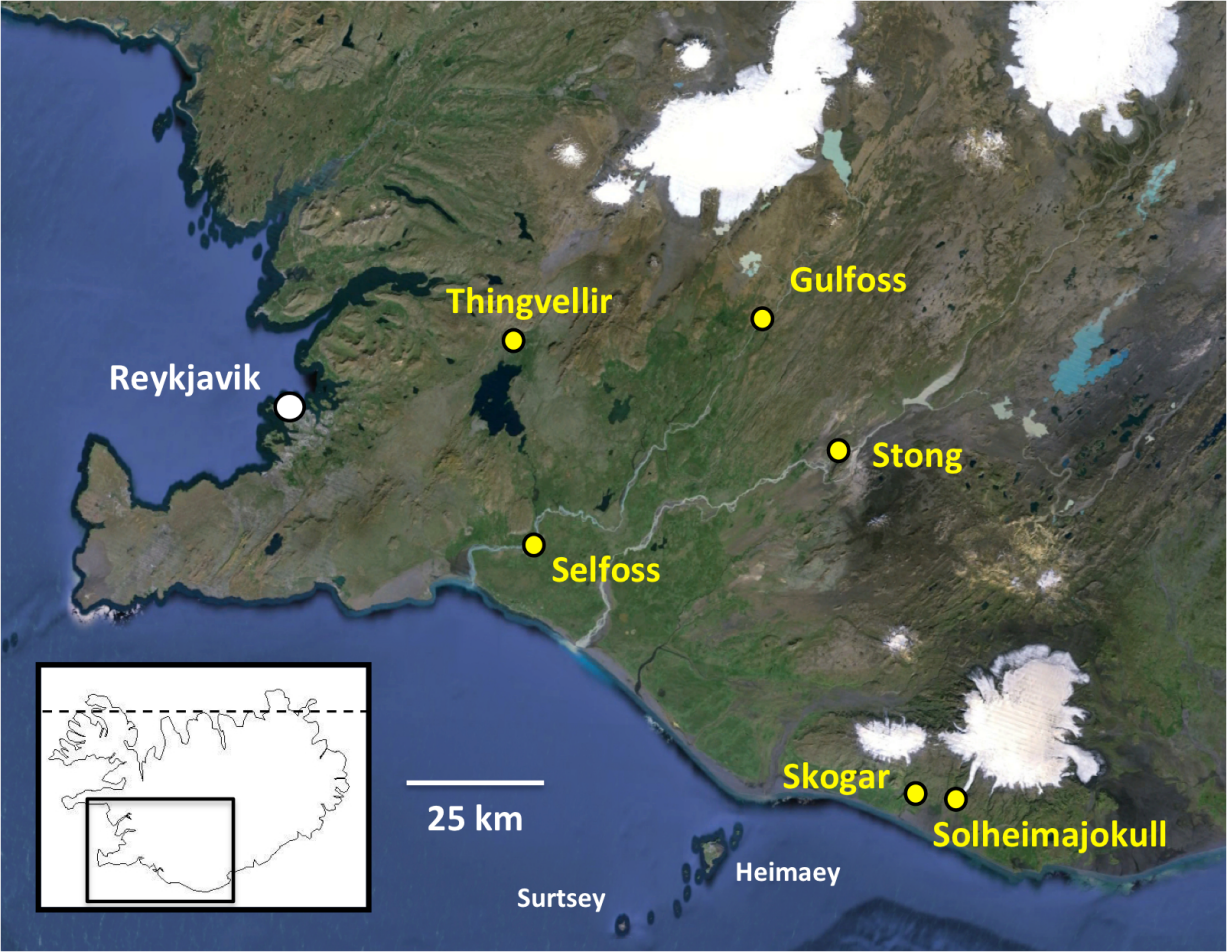
Populations of *Platanthera hyperborea* sampled in southwest Iceland during the present study. Base image also shows the capital Reykjavik, southwestern islands, icecaps and major rivers (courtesy of GoogleEarth). The dashed line in the inset denotes the Arctic Circle.

Initially, taxonomic discussions were driven entirely by perceived phenotypic complexity (cf. Luer, 1975; Sheviak, 2002; Delforge, 2006), but later became informed by the gradual accumulation of molecular systematic data across the genus (Hapeman & Inoue, 1997; Bateman et al., 2003; Bateman et al., 2009). More focused investigations of North American representatives of Section *Limnorchis*, most notably those pursued by Lisa Wallace, have revealed considerable genetic complexity, driven in part by allopolyploidy (Wallace, 2002; Wallace, 2003; Wallace, 2004; Wallace, 2006; reviewed by Bateman et al., 2009). One of the many significant implications of this work is that most studies of any kind that purport to have involved *Platanthera hyperborea* may not in fact have done so; rather, most have investigated materials that originated from North America or, less frequently, northeast Asia and are therefore likely to represent segregates of *P. hyperborea s.s*. The holotype of ‘*Orchis*’ (later *Platanthera*) *hyperborea* bears the label “Oxeraa, Iceland, 1767.” We presume that this is a reference to the River Oxeraa, which runs through the historical capital of Iceland at Thingvellir in southwest Iceland (Fig. 1). Given that it was the first member of Section *Limnorchis* to be formally described, Icelandic *P. hyperborea* is inevitably pivotal in unravelling the systematics of the group. However, no Icelandic plants have yet been analysed in order to test some of the intriguing hypotheses that have emerged from the North American research, not least the possibility that the lineage migrated as airborne seed to Iceland eastward from North America (most likely via southern Greenland).

Our recent work on *Platanthera* section *Platanthera* emphasised the severe threats to, and need for improved conservation of, the two rarer species endemic to the Azorean archipelago (Bateman, Rudall & Moura, 2013; Bateman et al., 2014). A similar statement can be made with regard to *P. holochila* (Hillebr.) Kraenzlin in the Hawaiian islands (e.g., Torres-Santana, Bruegmann & Zablan, 2007); with only *ca* 30 plants surviving in the wild, this species should surely be designated as Critically Endangered, though it has not yet been formally assessed by the IUCN. Happily, no such conservation challenges exist with *P. hyperborea*, which is by far the most frequent orchid occurring on Iceland (e.g., Kristinsson, 2010, p. 174). We suspect that its ecological success at least partly reflects its exceptionally high frequency of seed-set, which has been widely hypothesised to have been enhanced through self-pollination leading to autogamy (e.g., Gray, 1862; Hagerup, 1952; Reinhard, 1977; Catling, 1983; Catling, 1990; Sheviak, 2000; Sheviak, 2002; Claessens & Kleynen, 2011). However, some aspects of its floral morphology are unusual, and its mode of pollination remains the subject of ongoing debate.

The present paper focuses on the results of a detailed field-based morphometric survey of populations attributed to *P. hyperborea* in SW Iceland, supported by microscopic examination of the flowers and bracts, and DNA sequences from each study population. We use the accumulated data to test previous morphology-based assertions that *P. hyperborea* is:

(1) a member of *Platanthera* Section *Limnorchis*;
(2) a phylogenetically derived product of an eastward (most likely post-glacial) migration of the lineage derived from a North American ancestor;
(3) a *bona fide* species closely related to, but reliably distinguishable from, the North American segregates of *P. hyperborea* (and, if so, whether any taxonomic and/or ecological structure can be detected among Icelandic populations);
(4) at least facultatively autogamous and cleistogamous, and whether it may also benefit from insect pollinators, potentially including mosquitoes.
  We also consider the broader implications of this study for:
(5) using morphometric datasets to circumscribe species versus supraspecific taxa;
(6) quantifying developmentally mediated functional constraints on (a) the relative sizes of floral organs and (b) the absolute minimum flower size likely to preserve effective reproductive function;
(7) inferring potential advantage to the species through evolving particular elements of an autogamous mode of reproduction.

## Materials and Methods

### Field sampling

**Figure 2:**
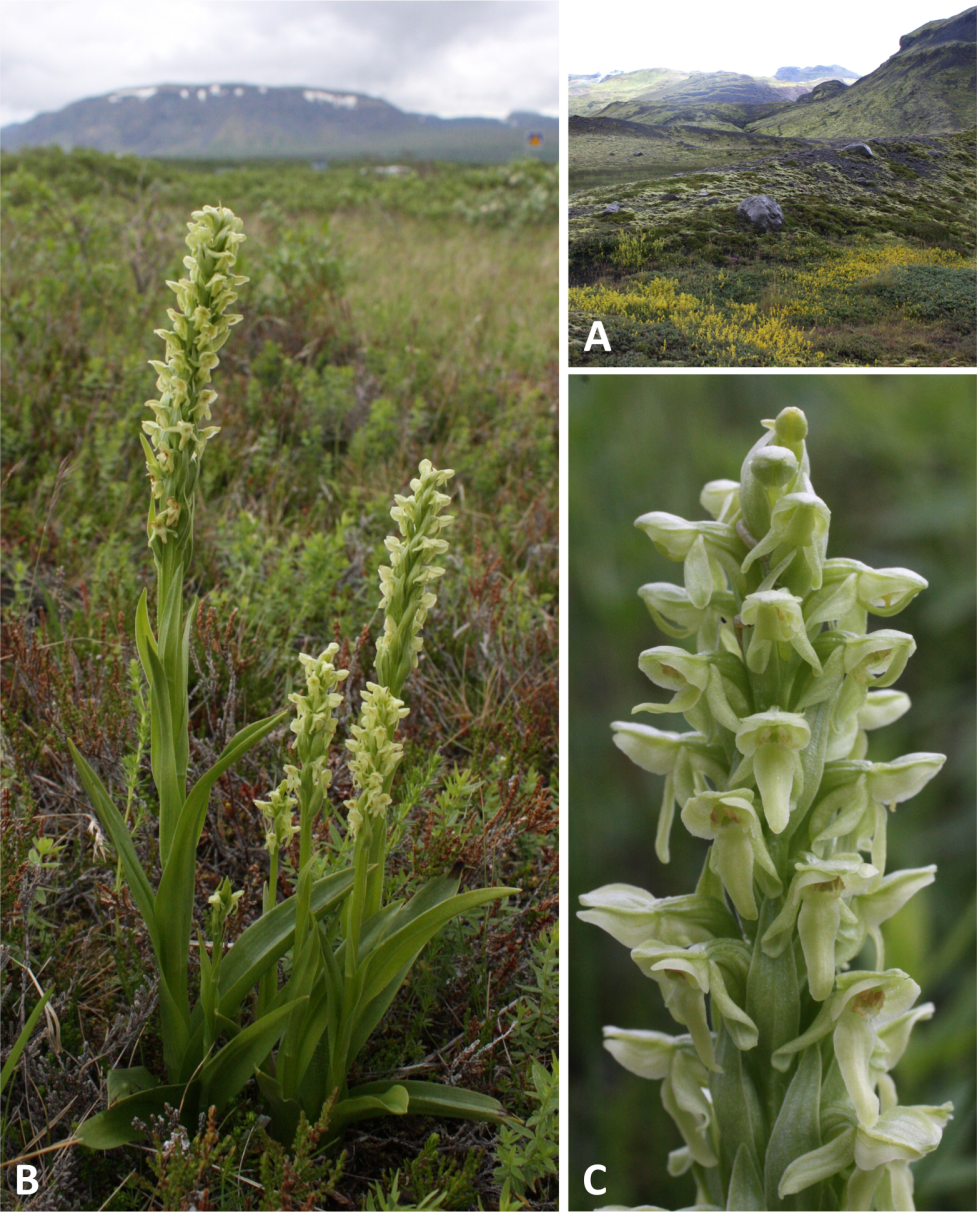
Classic plants and habitats of *Platanthera hyperborea* on Iceland. (A) The exposed orchid habitat close to the valley glacier at Solheimajokull. (B, C) Typical plants growing in the less exposed dwarf-scrub habitat at Thingvellir. Images: R Bateman.

**Figure 3:**
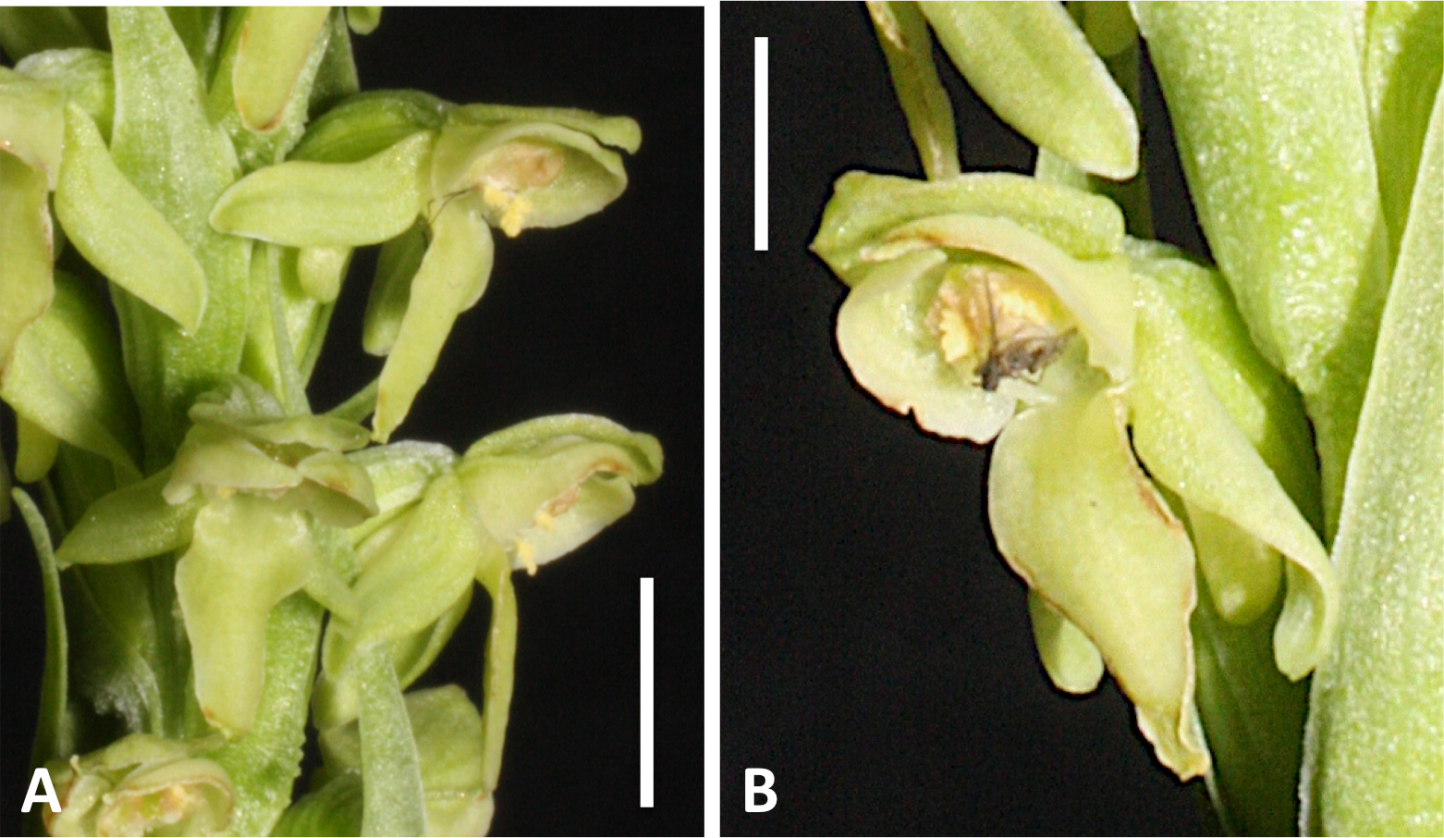
Flowers of an inflorescence of *Platanthera hyperborea* from Thingvellir. (A) Recently opened flowers showing that the pollen masses have already collapsed onto the stigma. (B) Flower at a later stage of anthesis with a mosquito glued to its stigma. Scale bar = 5 mm (A), 2.5 mm (B). Images: R Bateman.

RB and PR conducted a week-long field trip in the southwest quadrant of Iceland during the period 3rd–9th July 2014, ranging for approximately 140 km to the east and northeast of Reykjavik (Fig. 1). Our visit was a little later than ideal, flowering of the orchid having peaked an estimated ten days earlier (Table 1). Many populations of *P. hyperborea* were encountered. The six populations subjected to detailed study were chosen to represent wide ranges of geographic locations, habitat types and population sizes (Fig. 1, Table 1); they included the presumed *locus classicus* of the species at Thingvellir. Ten plants in each population were selected for detailed measurement where feasible (only six plants were found in measurable condition at Solheimajokull). One or two plants per population were also sampled for DNA analysis (a further DNA sample was collected at Geysir, near the study population at Stong), and at both the Selfoss and Thingvellir populations, multiple inflorescences were removed for microscopic study. In addition, representative individuals from several populations were imaged *in situ* (Figs. 2 and 3).

**Table 1:** Details of the six Icelandic localities sampled for *P. hyperborea* during early July 2014.

Locality	Latitude and longitude	Habitat	Altitude (m asl)	Peak flowering	Samples taken
THINGVELLIR	64°16′57.18″–21°05′21.67″	SG	140	6/3–4	M10*, P3, S1
SELFOSS	63°56′18.21″–21°00′15.24″	SG	4	6/4	M10, P3, S2
GULFOSS	64°19′10.30″–20°08′06.70″	SG	200	6/4	S1
STONG	64°07′28.54″–19°49′40.33″	HT	160	6/4–7/1	M10, S2
SKOGAR	63°31′43.94″–19°30′48.51″	SH	30	6/4	M10, S2
SOLHEIMAJOKULL	63°31′39.35″–19°22′06.72″	HT	110	6/4–7/1	M6, S2

**Notes.**

Habitat: HT, heathy tundra; SG, open, low-growing *Salix*-dominated scrub and grassland; SH, scrubby heathland. Peak flowering estimates: the slash is preceded by the month and succeeded by the week(s) of that month. Materials/data gathered (parenthetic figures are the number of samples acquired): M, morphometrics (asterisk indicates flowers decayed before morphometric measurements could be taken); P, spirit-preserved inflorescences; S, silica-gel samples of flowers for DNA analysis.

Our within-site sampling strategy for morphometric measurements was designed to minimise disturbance to individual plants. Destructive measurements of tubers were not attempted. Within each population, plants for study were chosen to proportionately reflect the range of variation evident in both morphology and habitat. Vegetative characters were measured non-destructively from *in situ* plants, and only approximately five flowers from each plant were removed for further study: one was permanently mounted and measured, whereas the remainder were placed in fine-grained dried silica gel to act as a DNA-friendly voucher. Wherever feasible, the florets chosen to provide morphometric data on the flower, ovary and bract were located 30–40% of the distance from the base to the apex of the inflorescence, in order to minimise the taxonomically widespread effect of diminution in flower size toward the apex. However, it proved necessary to sample higher in the inforescence in plants from populations where anthesis was relatively advanced.

### Morphometrics

#### Characters and matrices

Our previous studies of *Platanthera* section *Platanthera* (Bateman, James & Rudall, 2012; Bateman, Rudall & Moura, 2013; Bateman et al., 2014) identified 38 characters to be scored morphometrically (listed as appendix 1 by Bateman, Rudall & Moura, 2013). One character (C4: pale green versus dark green pigmentation of the labellum) initially measured by Bateman et al. was subsequently judged to largely duplicate another character (C5: maximum extent of green pigmentation on the labellum) and was therefore omitted from all analyses. The surviving 37 characters described the stem and inflorescence (4), leaves (7), bracts (5), labellum (4), spur and ovary (5), sepals and lateral petals (5), and gynostemium (7). They could alternatively be categorised as metric (27), meristic (3), multistate-scalar (6), and operationally bistate (1). Metric characters for most floral organs were measured at a resolution of 0.1 mm using a Leitz × 8 graduated ocular, though the two floral bract-cell characters were recorded in µm at ×100 magnification under a Leica Dialux 20 compound microscope.

This previous spectrum of morphological characters formed the core of the present study, but required modest additions and amendments. The complete absence of anthocyanin pigments from Section *Limnorchis* rendered redundant our usual practice of quantitatively colour matching various flower-parts for each measured part. More significantly, the exceptionally small size, partial closure and uniform colour of the flowers of *P. hyperborea* precluded accurate measurement by eye of five metric characters used by us in previous studies of *Platanthera* species to document details of the gynostemium (C17–C21 of Bateman, Rudall & Moura, 2013, appendix 1). This meant that we were obliged to omit these characters from any analysis that was based only on individual plants of *P. hyperborea.* For our two comparisons with Section *Platanthera* we relied instead on measurements of these characters taken under the SEM from flowers derived from just five individuals of *P. hyperborea*. Also, for the species-level analysis that was based on mean values of characters, we added to the list of 37 usable characters inherited from our previous studies two further characters. Both characters described the presence and micromorphology of epidermal papillae, firstly located within the spur (C9A: Papillae absent [0]: present, rod-shaped [1]: present, club-shaped [2]) and secondly on the stigmatic surface (C21A: Stigmatic papillae absent [0]: present [1]). We also scored these characters retrospectively for species of *Platanthera* previously studied by us. In total, four morphometric matrices were compiled:

(1) A matrix of 36 individual plants of *P. hyperborea*, representing four populations (omitting the vegetative-only dataset from Thingvellir) and 28 variable characters—of the original 37 characters, five gynostemial characters (C17–C21) were not measured and a further four characters proved to be invariant. Specifically, all measured plants of *P. hyperborea* possessed uniformly green labella (C5) and lateral petals (C14A), had strongly forward-curved labellar spurs (C9), and produced expanded leaves that lacked well-developed petioles (C36). The resulting matrix contained only 0.6% of missing values.
(2) A matrix of five sets of character mean values describing the Icelandic populations of *P. hyperborea* (including Thingvellir: data summarised in Table 2), also based on 28 variable characters.
(3) A matrix of 406 individual plants that adds 36 plants of *P. hyperborea* to the matrix of 370 plants of seven putative species of Eurasian species within Section *Platanthera* and 37 variable characters that was previously published by Bateman, Rudall & Moura (2013).
(4) A matrix of eight sets of taxon mean values that adds *P. hyperborea* to seven putative species of Eurasian species within Section *Platanthera* previously published by Bateman et al. (2014). The original 37 variable characters were supplemented with two further characters describing papillae within the spur (C9A) and on the stigma (C21A).

**Table 2:** Population means, sample standard deviations (SSD) and coefficients of variation (CV, %) for 38 morphometric characters from five populations of *P. hyperborea*, followed by taxon mean values derived from the first four populations.

	Population	Length lip	Width lip	Reflexion lip	*Presence pigment lip*	*Extent pigment lip*	Length spur	Width mouth spur	Width halfway spur	*Curvature spur*	Length ovary
Mean	Selfoss	5.04	1.88	1.6	2	100	3.41	0.82	0.99	5	9.9
SSD		0.62	0.32				0.49	0.12	0.12		1.2
CV(%)		12	17				14	15	12		12
Mean	Stong	5.11	1.63	1.9	2	100	3.69	0.75	0.94	5	8.2
SSD		0.43	0.19				0.30	0.11	0.15		0.9
CV(%)		8	12				8	15	16		11
Mean	Skogar	4.81	1.91	1.5	2	100	3.88	0.74	0.88	5	9.0
SSD		0.31	0.24				0.25	0.11	0.09		1.2
CV(%)		6	13				6	15	10		13
Mean	Solheimaj.	5.14	1.87	1.7	2	100	3.66	0.94	0.86	5	9.3
SSD		0.51	0.16				0.26	0.21	0.09		0.8
CV(%)		10	9				7	22	10		9
Mean	Thingvellir	NM	NM	NM	NM	NM	NM	NM	NM	NM	NM
SSD											
CV(%)											
Mean	*hyperborea*	5.01	1.8	1.67	2	100	3.66	0.8	0.9	5	9.08
SSD		0.48	0.26				0.40	0.14	0.12		1.20
CV(%)		9.6	14.4				10.9	17.5	13.3		13.2

	Population	Pos. lateral sepal	Length lateral sepal	Width lateral sepal	Length lateral petal	*Colour lateral petal*	Length column	Width column	Width stigma	Length pollinaria	Distance viscidia
Mean	Selfoss	0.8	4.22	1.55	3.43	1	1.65	1.79	NM	NM	NM
SSD			0.64	0.22	0.29		0.18	0.2			
CV(%)			15	14	8		11	11			
Mean	Stong	0.5	4.12	1.57	3.6	1	1.62	1.72	NM	NM	NM
SSD			0.41	0.3	0.45		0.1	0.19			
CV(%)			10	19	13		6	11			
Mean	Skogar	1.4	4.38	1.69	3.28	1	1.67	1.68	NM	NM	NM
SSD			0.66	0.25	0.43		0.19	0.26			
CV(%)			15	15	13		11	15			
Mean	Solheimaj.	0.8	4.23	1.6	3.48	1	1.67	1.72	NM	NM	NM
SSD			0.28	0.22	0.32		0.1	0.2			
CV(%)			7	14	9		6	12			
Mean	Thingvellir	NM	NM	NM	NM	NM	NM	NM	NM	NM	NM
SSD											
CV(%)											
Mean	*hyperborea*	0.89	4.24	1.6	3.44	1	1.82	1.65	1.18	1.01	1.08
SSD			0.53	0.24	0.39		0.07	0.18	0.1	0.04	0.15
CV(%)			12.5	15	11.3		3.8	10.9	8.5	4.0	13.9


	Population	Distance pollin. apices	*Presence auricles*	Mean cell diam. bract	Mean cell shape bract	Width floral bract	Length floral bract	Length basal bract	Stature stem	Inflorescence length	No. flowers
Mean	Selfoss	NM	NM	90.6	2.9	2.41	9.2	14.5	16.6	50.4	20.4
SSD				19.2		0.32	1.9	3.2	6.6	20.1	11.3
CV(%)				21		13	21	22	40	40	55
Mean	Stong	NM	NM	81.8	2.9	2.59	9.6	12.9	16.7	40.5	15.7
SSD				7.5		0.30	0.8	3.7	4.6	17.2	11.4
CV(%)				9		12	8	29	28	42	73
Mean	Skogar	NM	NM	80.3	2.9	2.32	9.1	14.5	16.0	50.4	19.7
SSD				4.4		0.19	0.9	3.8	6.8	20.3	7.9
CV(%)				5		8	10	26	43	40	40
Mean	Solheimaj.	NM	NM	87.8	2.8	2.3	9.8	14.5	11.8	35.5	17.0
SSD				5.8		0.2	0.8	2.1	2.9	8.5	7.2
CV(%)				7		9	8	14	25	24	42
Mean	Thingvellir	NM	NM	NM	NM	NM	NM	13.5	12.5	43.3	25.0
SSD								2.5	3.1	13.8	9.3
CV(%)								19	25	32	37
Mean	*hyperborea*	0.45	0	84.8	2.89	2.42	9.39	14.06	15.67	45.17	18.33
SSD		0.05	0	11.8		0.28	1.2	3.29	5.72	18.31	9.67
CV(%)		11.1	0	13.9		11.6	12.8	23.4	36.5	40.5	52.4

	Population	Stem diameter	Non-sheathing leaves	No. sheathing leaves	Width longest leaf	Length longest leaf	Position max. width	*Petiole development*	Angle leaf vs ground
Mean	Selfoss	3.55	1.8	3.6	13.7	73.2	34.2	0	1.9
SSD		1.33			4.8	16.7	3.7		
CV(%)		37			35	23	11		
Mean	Stong	3.14	2.2	3.2	11.8	75.7	38.2	0	2
SSD		1.06			3.3	14.4	3.5		
CV(%)		34			28	19	9		
Mean	Skogar	3.63	2.4	3	13.2	68.0	35.8	0	2
SSD		1.34			3.7	19.0	3.6		
CV(%)		37			28	28	10		
Mean	Solheimaj.	3.3	1.8	3.5	12.8	62.3	33.5	0	2.5
SSD		0.7			2.4	10.6	3.9		
CV(%)		21			19	17	12		
Mean	Thingvellir	3.53	2.5	3.1	12.3	61.8	40.2	0	2.2
SSD		0.97			3	9.7	5.9		
CV(%)		27			24	16	15		
Mean	*hyperborea*	3.42	2.06	3.31	12.89	70.64	35.64	0	2.1
SSD		1.15			3.7	16.03	3.95		
CV(%)		33.6			28.7	22.7	11.1		

**Notes.**

Data reflect sets of ten plants except Solheimajokull (six plants). Asterisks indicate absence of data. Invariant characters are italicised.

#### Data analysis

Morphometric data for individual plants were summarised on an Excel v14.4 spreadsheet. Mean values, plus sample standard deviations and coefficients of variation for all metric and some meristic characters, were calculated for every character in each of the five populations of *P. hyperborea* as well as for the species as a whole. Univariate and bivariate analyses were summarised and presented using Deltagraph v5.6 (SPSS/Red Rock software, 2005), which in some cases was also used to calculate linear regressions.

Multivariate analyses were performed using Genstat v14 (Payne et al., 2011). For each of the four matrices, the selected characters were used to compute a symmetrical matrix that quantified the similarities of pairs of data sets (i.e., plants) using the Gower Similarity Coefficient (Gower, 1971) on unweighted data sets scaled to unit variance. The resulting matrix was in turn used to construct a minimum spanning tree (Gower & Ross, 1969) and subsequently to calculate principal coordinates (Gower, 1966; Gower, 1985)—compound vectors that incorporate positively or negatively correlated characters that are most variable and therefore potentially diagnostic. Principal coordinates are especially effective for simultaneously analysing heterogeneous suites of morphological characters and can comfortably accommodate missing values; they have proven invaluable for assessing relationships among orchid species and populations throughout the last three decades (reviewed by Bateman, 2001). For each multivariate analysis, dendrograms were generated from the Gower Similarity values and the first four principal coordinates (PCo1–4) were plotted together in pairwise combinations to assess the degree of morphological separation of individuals (and thereby of populations and taxa) in these dimensions. Pseudo-F statistics were obtained to indicate the relative contributions to each coordinate of the original morphometric variables.

### Microscopic examination and imaging

Small numbers of inflorescences, preferring those still retaining a proportion of unopened buds, were sampled from populations in contrasting habitats at Selfoss and Thingvellir and stored in 70% ethanol. Preparation for scanning electron microscopy (SEM) involved selecting flowers from each inflorescence for dehydration through an alcohol series to 100% ethanol. They were then stabilised using an Autosamdri 815B critical-point drier, mounted onto stubs using double-sided adhesive tape, coated with platinum using an Emtech K550X sputter-coater, and examined under a Hitachi cold-field emission SEM S-4700-II at 2 kV. The resulting images were recorded digitally for subsequent manipulation in Adobe Photoshop.

### Phylogeny reconstruction

#### Data acquisition

Total genomic DNA was extracted from silica-desiccated floral (or, less often, leaf) material using the standard 2× CTAB procedure (Doyle & Doyle, 1990) except that extractions were incubated in 500 µl CTAB buffer, 50 µl sarkosyl and 10 µl proteinase-K. The rapidly mutating ITS region of nuclear rDNA (e.g., Baldwin et al., 1995) was amplified in full (ITS1–5.8S–ITS2) via polymerase chain reaction (PCR) using the primers, reaction mixture and cycling parameters detailed by Sramkó et al. (2014). The successfully amplified PCR products were then transferred to Macrogen Inc., South Korea for cleaning and sequencing. Sequencing used the dideoxy chain-terminating method, employing the same primers used for PCR amplification.

#### Data analysis

The majority of the 150 ITS sequences used for phylogeny reconstruction were inherited from previous studies (Bateman et al., 2009; Bateman et al., 2014; Bateman, James & Rudall, 2012). The samples span four closely related genera, and include representatives of all but one taxonomic section with the genus *Platanthera*. Sampling is especially dense for Section *Limnorchis* (the focus of the present study) and Section *Platanthera*, the latter encompassing the following taxa: *P. bifolia* (18 sequences), *P. chlorantha* (22), their hybrid (3), *P. holmboei* (7), *P. algeriensis* (3), *P. pollostantha* (25), *P. micrantha* (9), their hybrid (2), and *P. azorica* (3). The main purpose of the present analysis was to include for the first time six new accessions of *bona fide P. hyperborea* from Iceland, which were placed in the context of an additional 17 ITS sequences, downloaded from GenBank and ostensibly derived from *P. dilatata* vars. *dilatata* (5), *albiflora* (2) and *leucostachys* (2), *P. huronensis* (1), and *P. aquilonis* (7). Three of the sequences were incomplete: single identical sequences attributed to each of *P. aquilonis* and *P. huronensis* by Kuzmina et al. (2012) encompassed only ITS2, whereas our own sample of Icelandic *P. huronensis* from Selfoss could be read only for the bulk of ITS1 due to the presumed presence of a second, length-polymorphic copy of ITS (cf. Sramkó et al., 2014). Single exemplars only of each detected ribotype were carried forward to the parsimony analysis, in order to simplify the tree-building procedure.

#### Tree-building methods

Together, the 150 accessions generated 46 ITS ribotypes representing 40 named ingroup taxa plus the two outgroup species of *Pseudorchis*. In order to accelerate the tree-building procedure, and to facilitate full rather than fast bootstrap analysis, trees were constructed only from these 46 ribotypes, rather than from all 150 plants. Alignment was achieved by eye, yielding a total of 654 usable positions. All indels were coded as bistate characters, subsequently treating the actual gaps as missing values. Each differentiable gap of equal length was coded separately, thereby maximising the number of indels recognised (58) but also maximising the proportion of those indels that functioned only as *de facto* autapomorphies unique to individual plants (36, = 62%). Trees were constructed in PAUP v4.0b10 (Swofford, 2001). *Pseudorchis* was identified as the outgroup genus, following the topology of Bateman et al. (2003). Parsimony trees were generated via heuristic search using subtree pruning-regrafting (SPR) with MulTrees in effect, no limit on number of trees held, and swapping on all trees—a protocol designed to recover all islands of most-parsimonious trees. Topological corroboration was sought through a maximum likelihood analysis of the same matrix using the default parameters. With this exception, we considered it unnecessary to repeat during the present study the wider range of analytical experiments conducted on the original matrix by Bateman et al. (2009).

Statistical robustness of nodes in parsimony tree-sets was explored via full bootstrap analyses using a full heuristic search with stepwise addition, permitting 1000 replicates. The comparatively large numbers of suboptimal trees found in the primary analysis under collapse nodes with minimum length of zero under the amb- setting limited calculation of Bremer support (decay index) values to two or less.

## Results and Discussion

### Molecular phylogenetics

**Figure 4:**
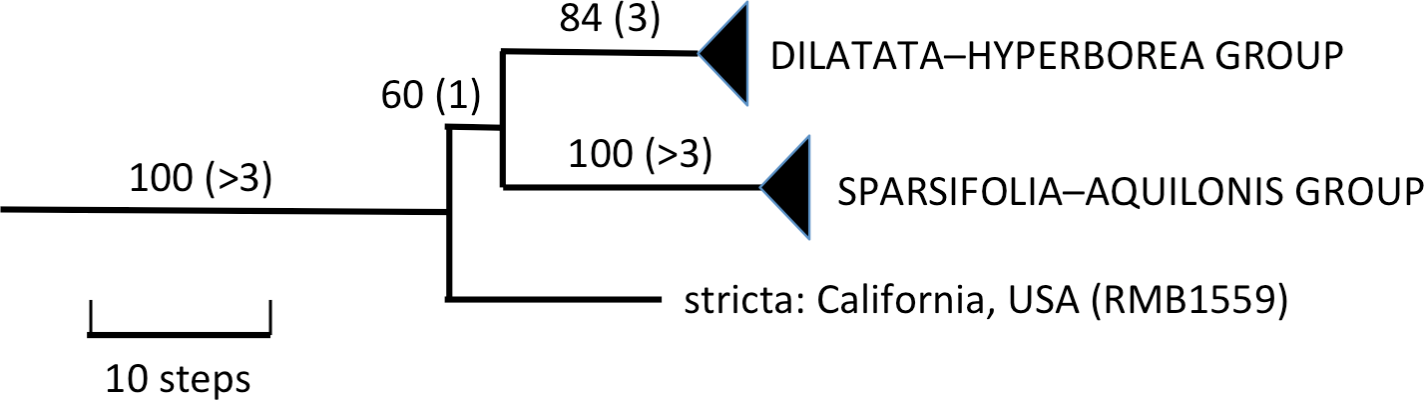
Portion of the preferred most-parsimonious ITS tree that encompasses *Platanthera* section *Limnorchis*, showing three well-supported species groups. Figures are bootstrap values and (in parentheses) decay indices.

The 46 ribotypes included in the aligned ITS matrix yielded 713 characters (including 58 scored indels), of which 197 (including 22 indels) proved phylogenetically informative. When analysed under amb- nodal collapse in PAUP, the matrix yielded 625 most-parsimonious trees of length 691 steps, Consistency Index of 0.645 (0.546 when excluding mutations unique to a single ribotype, i.e., autapomorphies *sensu lato*) and Retention Index of 0.826. Unsurprisingly, adding a further seven low-divergence ITS ribotypes for *P. hyperborea* and its close relatives did not alter the broad-brush topologies that we have generated during previous analyses of ITS data in *Platanthera* (Bateman et al., 2009; Bateman et al., 2014). Rather than reproduce the entire *Platanthera* phylogeny yet again, here we have chosen to illustrate only the relevant clade within the genus, Section *Limnorchis* (Fig. 4).

The Icelandic populations of *P. hyperborea* are placed unequivocally within the *dilatata-hyperborea* group of the first-divergent section of *Platanthera s.l.*, Section *Limnorchis*. Section *Limnorchis* is strongly supported as monophyletic and in Fig. 4 forms a near-trichotomy of three well-supported groups: *P. stricta* from western North America, the *sparsifolia-aquilonis* group (also North American), and the *dilatata-hyperborea* group, centred on North America but more geographically widespread. Our ITS trees tentatively suggest that our Californian sample of *P. stricta* is sister to the two remaining groups (Fig. 4), whereas the two Montanan samples of *P. stricta* analysed by Wallace (2002) placed as sister to the *P. dilatata-hyperborea* group in her ITS tree (albeit without bootstrap support).

**Figure 5:**
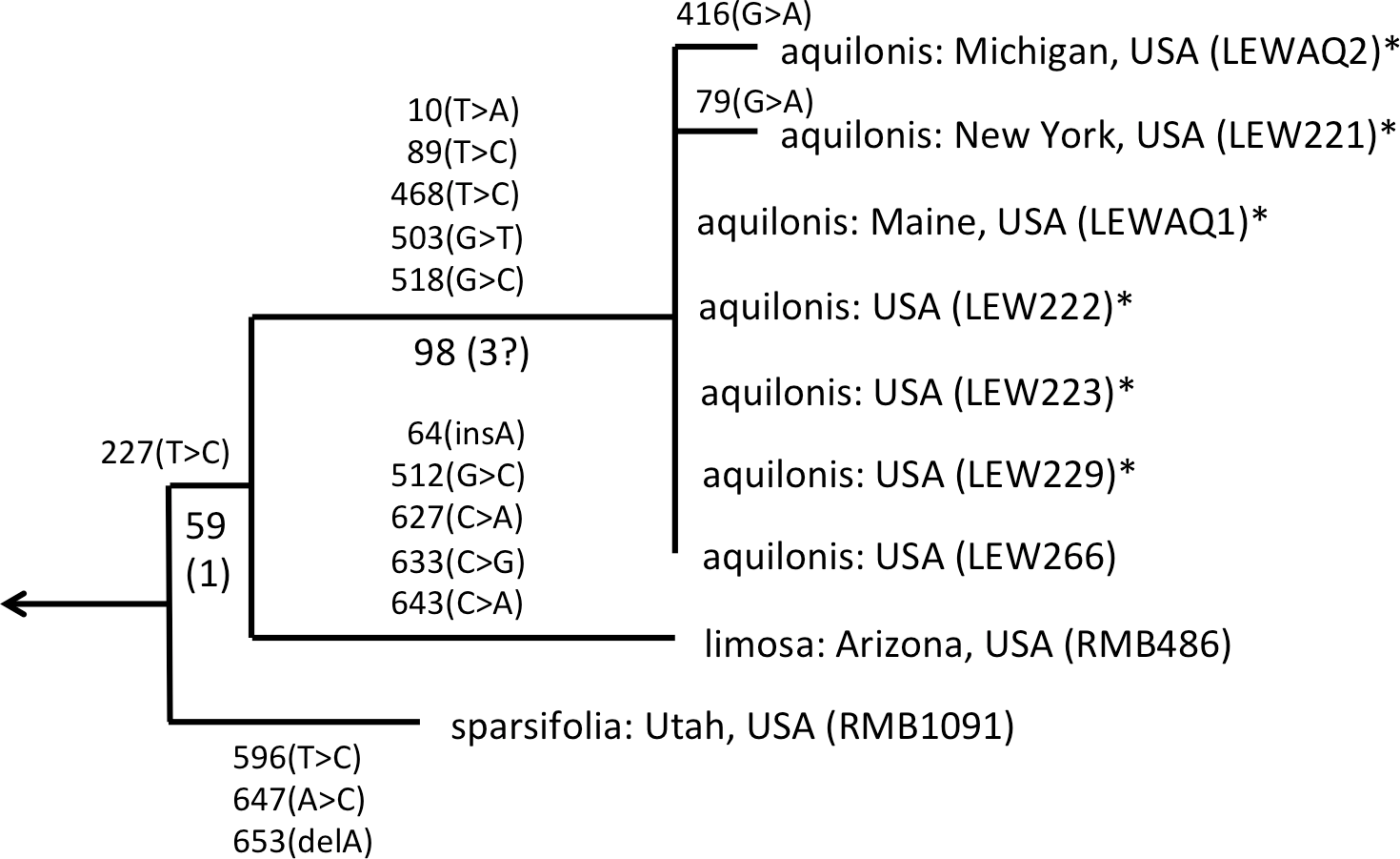
ITS phylogeny of the *P. sparsifolia–aquilonis* group enlarged from Fig. 4 to show the molecular character-state transitions that separate the nine analysed accessions. Figures are bootstrap values and (in parentheses) decay indices.

As observed by Wallace (2002) and Bateman et al. (2009), the well-supported *sparsifolia-aquilonis* group consists of a near-trichotomy; the paraphyletic pairing of southwestern *P. sparsifolia* and *P. limosa* apparently gave rise to the more geographically widespread, more northerly and typically less vegetatively robust, smaller-flowered *P. aquilonis* (Fig. 5) (cf. Luer, 1975; Sheviak, 2002). Inclusion in our matrix of seven plants of *P. aquilonis* analysed for ITS by Wallace (2002) and Wallace (2004) indicates the existence of a widespread plesiomorphic ribotype that has generated at least two more derived ribotypes through mutation of single bases.

**Figure 6:**
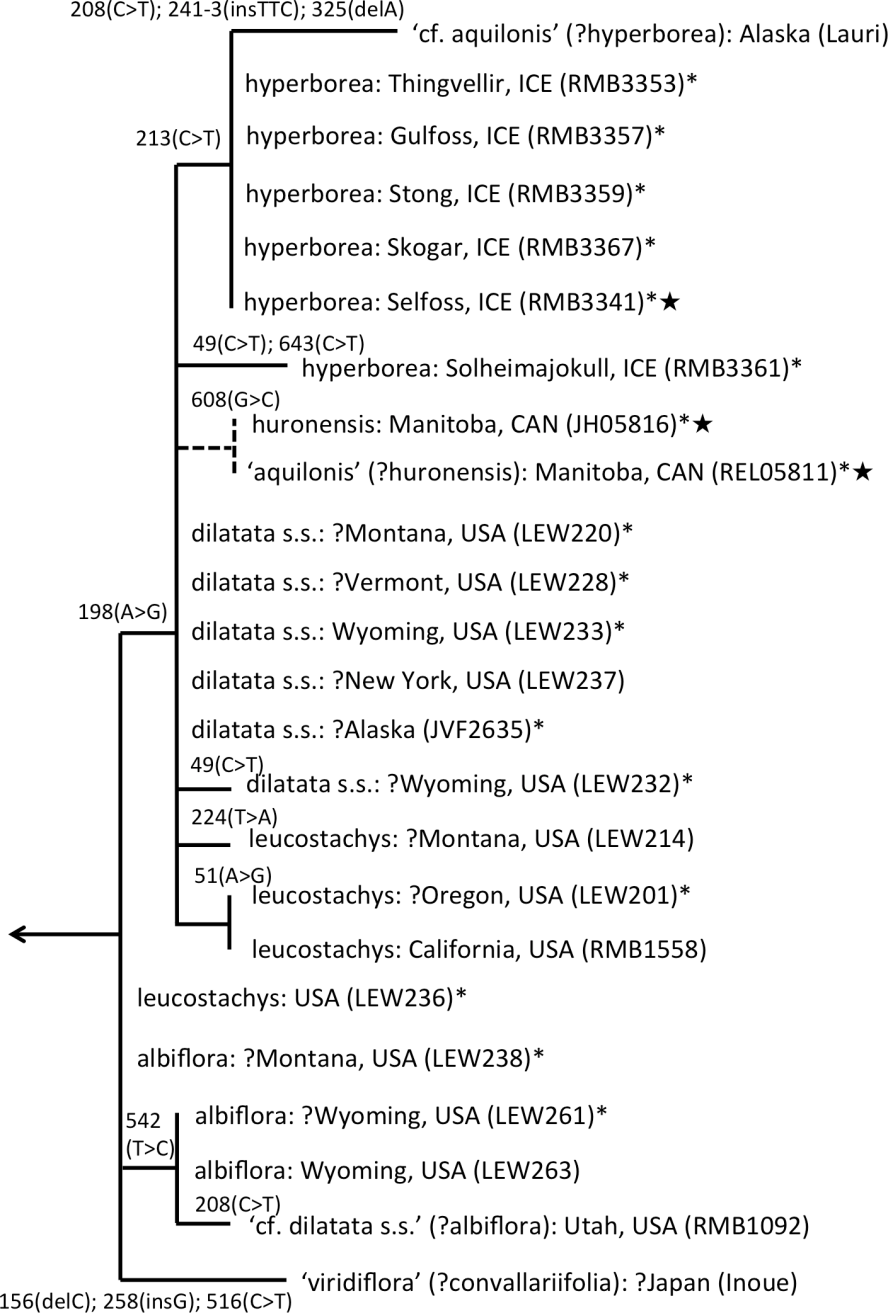
ITS phylogeny of the *P. dilatata–hyperborea* group enlarged from Fig. 4 to show the molecular character-state transitions that separate the 24 analysed accessions. Asterisked sequences are new to this analysis, starred sequences are only partial. All branches were too short to receive bootstrap support.

The main focus of the present study is the equally well-supported, but taxonomically highly controversial, *dilatata-hyperborea* complex (Fig. 6). The traditional classification devised by Sheviak (2002) recognised several species in North America. These included *P. dilatata* (including two varieties occurring only along the western seaboard—*albiflora* and *leucostachys*), the similarly distributed *P. huronensis*, and *P. convallariifolia* and *P. hyperborea*—two species morphologically similar to *P. huronensis* and believed by Sheviak (2002) to be confined within the North American region to Alaska and Greenland, respectively. According to Sheviak, *P. convallariifolia* occurs primarily in Japan and Kamchatka, and *P. hyperborea* primarily in Iceland. Although he attributed most geographically intervening Canadian and American populations of similar morphology to *P. aquilonis*, we have seen from ITS evidence that these plants belong to a clade that is both separate and distinct from *P. aquilonis* (Figs. 4 and 5).

ITS divergence within the *dilatata-hyperborea* group is low (Fig. 6), the maximum divergence being the eight characters (four SNPs and four indels: 1.1% divergence) that separate two samples of suspect identities: a Japanese sample of ‘*viridiflora*’ and an Alaskan sample of ‘*aquilonis*.’ If taken at face value, the ITS data do not appear to add greatly to our understanding of species circumscription within the *dilatata-hyperborea* group, let alone elucidate the phylogenetic relationships of named taxa; formal Linnean epithets superficially appear to be distributed near-randomly across the tree (Fig. 6).

However, we hypothesise that, given the notorious phenotypic similarity of these taxa, the relatively poor fit between ribotype and Linnean epithet partly reflects several misidentifications of plants sampled for DNA analyses by several researchers. These tentative, molecularly-driven re-identifications of molecular accessions require some modifications to the discussion of ITS patterns within the group previously offered by Bateman et al. (2009). Specifically, we suspect that the Japanese accession originally described as *P. hyperborea ‘viridiflora’* is actually attributable to the sole Asiatic member of Section *Limnorchis*, *P. convallariifolia*. The plant identified as *P. dilatata* var. *dilatata* collected by one of us (RB) in Utah may have been more appropriately assigned to *P. dilatata* var. *albiflora*. *Bona fide P. aquilonis* accessions have proved to be so divergent from *P. hyperborea* in ITS (*ca* 4%: Fig. 4) that the presence in the *P. hyperborea* clade of two plants originally attributed to *P. aquilonis* (Fig. 6) appears highly improbable; it seems to us far more likely that the plant collected in Manitoba by Royce Longton in 1971 and used to epitomise *P. aquilonis* in a subsequent herbarium-based DNA barcoding exercise (Kuzmina et al., 2012) was actually *P. huronesis*, a species that owes its origin to allopolyploidy between *P. aquilonis* and *P. dilatata s.l.* (Sheviak, 2002; Wallace, 2002; Wallace, 2003; Wallace, 2004; Wallace, 2006). Lastly, the specimen of *‘P. aquilonis’* from Alaska provided to us by Robert Lauri shares a synapomorphic mutation in ITS1 with most of our Icelandic samples of *P. hyperborea*, suggesting that *bona fide* populations of this species could conceivably occur much further west than some recent observers have suggested (e.g., Sheviak, 2002).

Interesting biogeographic patterns emerge from Fig. 6 irrespective of taxonomic assignment, but if our speculations regarding misidentification are accepted, a clearer *taxonomic* pattern also emerges from the ITS tree. The apparently most plesiomorphic ribotypes occur in single accessions of two western American segregates of *P. dilatata*, var. *albiflora* and var. *leucostachys*. From this origin emerged three more derived lineages that ostensibly (1) generated the remainder of var. *albiflora*, (2) migrated north-westward across the Bering Straits to invade northeast Asia as *P. convallariifolia*, and (3) generated the remaining taxa. The Group 3 lineage began as *P. dilatata* var. *dilatata*; populations possessing identical ribotypes occupy the northern montane regions of both western (including Alaska) and eastern North America. The clade later diversified to generate the remaining populations of var. *leucostachys* on the one hand and *P. hyperborea s.s.* on the other, implying migration to the north and east. Whether the molecularly distinctive Alaskan plant apparently derived from this core *dilatata-hyperborea* ribotype (mainly through indels: Fig. 6) should be assigned to *P. hyperborea* remains a moot point, requiring much more detailed comparative study of the morphology, karyology and genetics of populations of the *P. hyperborea* group in Greenland, Arctic Canada, Alaska and Kamchatka. In addition, the basic Group 3 ribotype was presumably donated through allopolyploidy to the tetraploid *P. huronensis* sequences from Manitoba that we included in our analysis, after which they acquired a further unique single-nucleotide polymorphism.

A more detailed molecular investigation of *P. huronensis* across North America conducted by Wallace (2002) and Wallace (2003) suggested that it has at least two separate allopolyploid origins (i.e., it is diphyletic); both RFLP and ITS data imply that western populations had *P. dilatata s.l.* as maternal parent, whereas eastern populations had *P. aquilonis* as maternal parent. Indeed, Wallace (2002, her Fig. 4.8) found ribotypes of *P. huronensis* plants to be scattered throughout the *dilatata-hyperborea* and *sparsifolia-aquilonis* groups.

**Figure 7:**
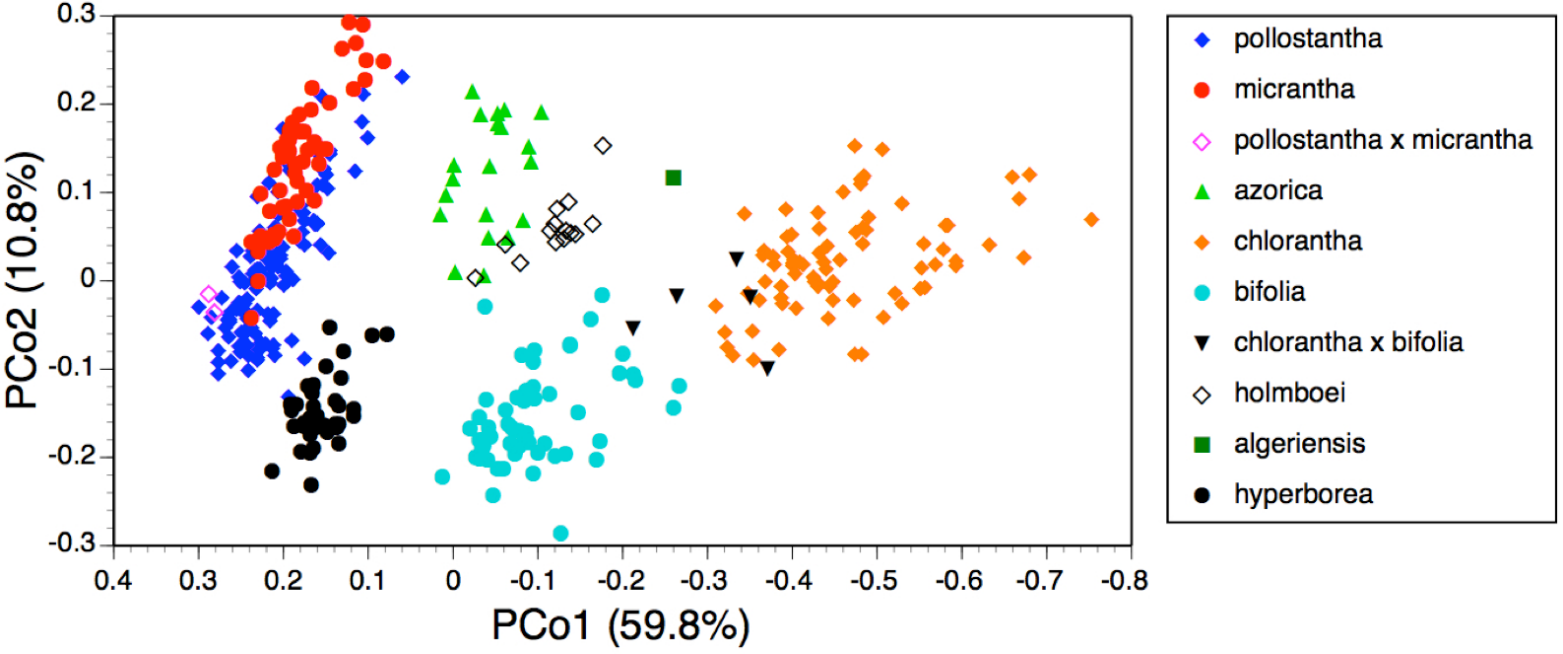
Bivariate scatter-diagram of the first two principal coordinates derived from 37 morphometric characters measured in individuals of *Platanthera hyperborea* plus seven putative species and two hybrid combinations in *Platanthera* section *Platanthera*. Parenthetic figures indicate the percentage of the total variance accounted for by each axis.

One final observation can be extracted from the ITS tree shown in Fig. 6. ITS sequences of Icelandic *P. hyperborea* from the Thingvellir, Gulfoss, Stong and Skogar populations were identical, but all differed from the ITS sequence from Solheimajokull in three C>T SNPs (representatives of these two ribotypes, from Solheimajokull and Stong, have been deposited in GenBank as KR074429 and KR074430, respectively). The absence from the Solheimajokull population of the C>T SNP at ITS1-213 suggests an independent origin of this ribotype from within *P. dilatata s.l*., though obviously, ITS cloning of a larger number of individuals would be required to strengthen this tentative interpretation. Morphologically, however, Solheimajokull is actually the least internally variable (Fig. 10) and least deviant (Fig. 7) of all the *P. hyperborea* populations that we examined.

### Morphology: multivariate analyses

**Figure 8:**
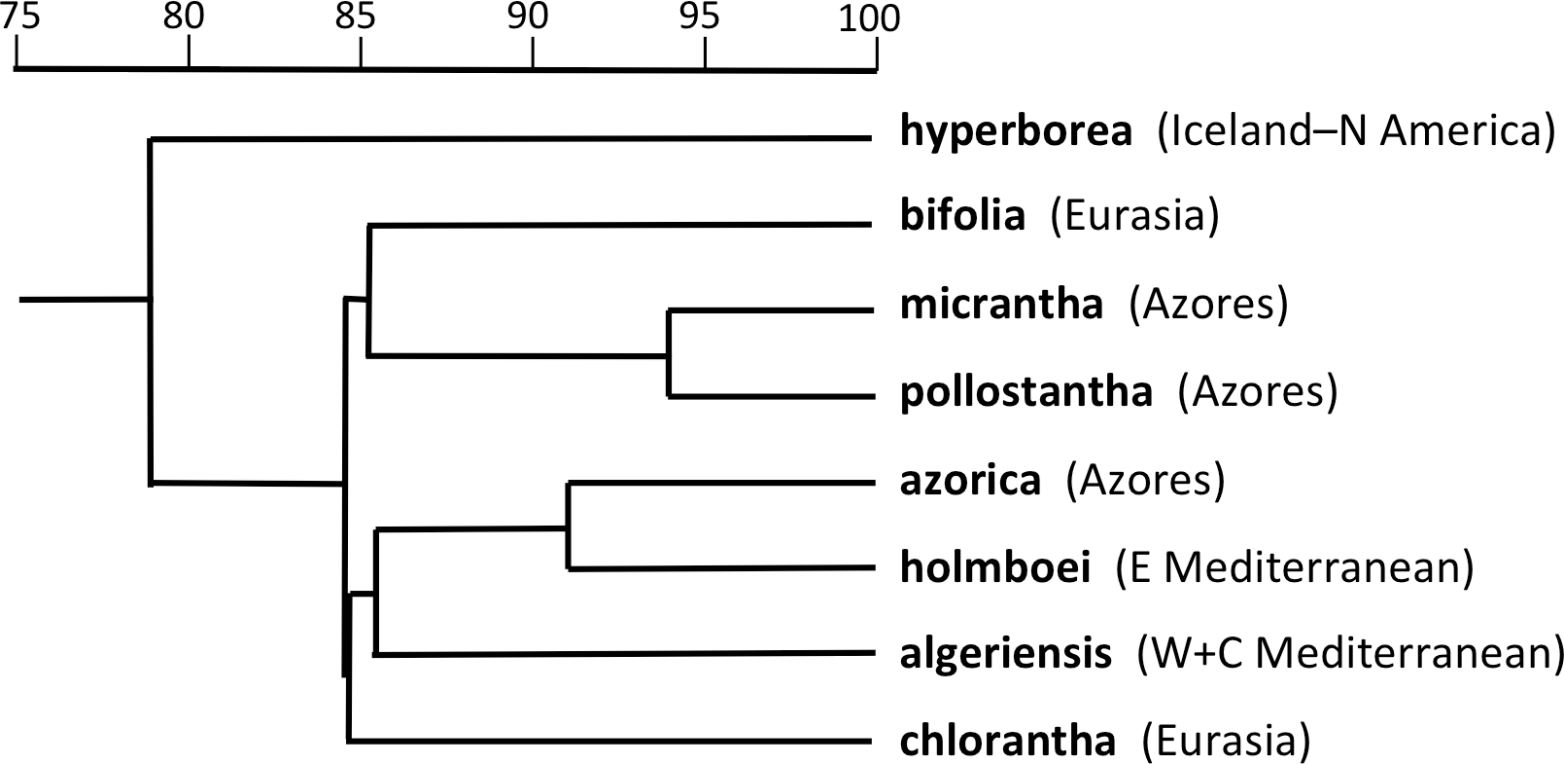
Dendrogram providing a morphometric comparison of Icelandic *P. hyperborea* with three Azorean and four Eurasian species of *Platanthera* section *Platanthera*. Values are Gower similarity coefficients based on taxon mean values for 39 variable characters.

The PCo plot of individual plants shown in Fig. 7 and the Gower similarity dendrogram of taxon mean values shown in Fig. 8 were intended primarily to explore how *P. hyperborea* compares in detailed morphology with the seven putative species of Section *Platanthera* previously analysed by us.

In the case of the PCo analysis (Fig. 7), the plot differs little from that previously generated from the seven species of Section *Platanthera* (Fig. 3 of Bateman et al., 2014). The 36 plants of *P. hyperborea* are simply interpolated into a formerly unoccupied region of hyperspace between the small-flowered Azorean endemics *P. pollostantha* and *P. micrantha* on the one hand and the Eurasian *P. bifolia* on the other. The only consequences for the previously inferred inter-species relationships are that *P. bifolia* is brought a little closer to *P. azorica* and *P. holmboei* on PCo2 and the percentage of total variance accounted for by PCo1 is reduced by 3% (though it remains exceptionally high at 60%). The *P. hyperborea* cluster is comparatively compact, scoring similarly to *P. micrantha* on PCo1 and to *P. bifolia* on the much weaker PCo2. The yet weaker PCo3 separates *P. hyperborea* from *P. bifolia* on the basis of the frequently petiolar leaves and white lateral petals of the latter (a feature shared with *P. chlorantha*).

Adding *P. hyperborea* (Fig. 8) caused modest modifications relative to the equivalent species-level dendrogram presented as their Fig. 6 by Bateman et al. (2014). Specifically, its inclusion increased the inferred similarity of the two small-flowered Azorean species, *P. pollostantha* and *P. micrantha*, but reduced the similarity of *P. chlorantha* to *P. algeriensis* such that *P. algeriensis* became perceived as being more similar to the pairing of *P. holmboei* plus *P. azorica*. Interestingly, the dendrogram concurred with the molecular phylogeny in showing *P. hyperborea* to have diverged prior to the species of Section *Platanthera*, the majority of which then separate over a remarkably short range of Gower similarities (84.5–85.6%). Examination of the underlying Gower similarity matrix showed that *P. hyperborea* had the strongest overall similiarities with *P. pollostantha* (79.0%) and *P. bifolia* (78.8%) but the weakest similarity with *P. chlorantha* (a remarkably low 41.3%).

**Figure 9:**
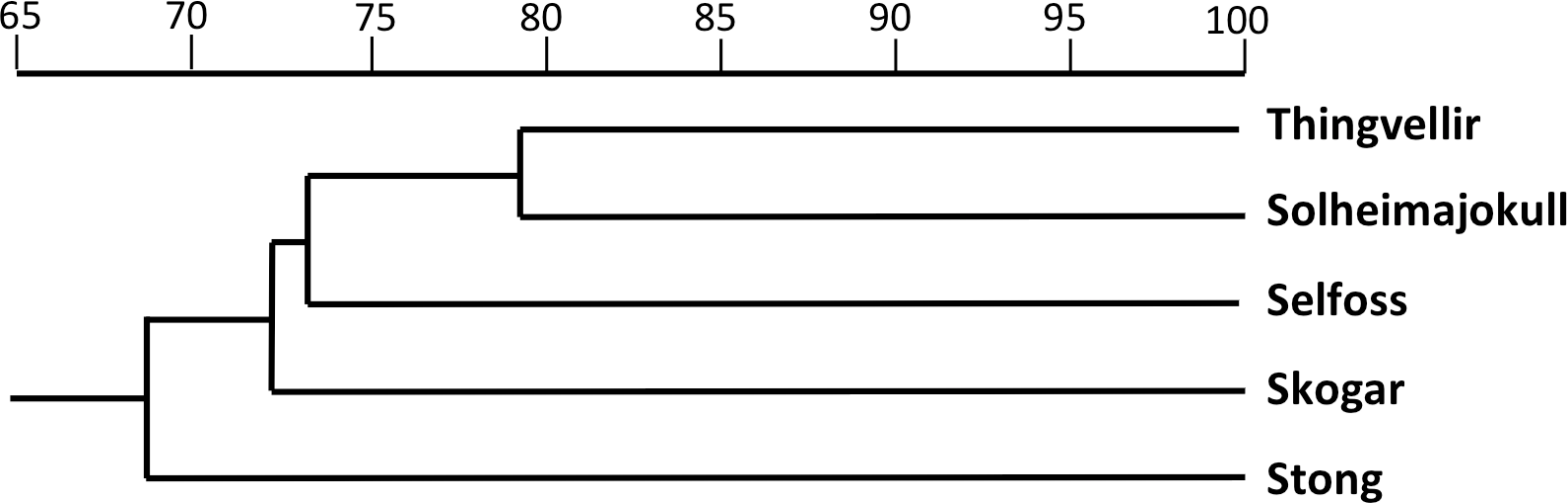
Dendrogram showing morphometric relationships of the five study populations of *P. hyperborea*. Values are Gower similarity coefficients based on population mean values for 28 varable characters.

The equivalent dendrogram based on mean values for populations of *P. hyperborea* only (Fig. 9) also shows the populations diverging over a relatively narrow range of similarities (68.5–73.3%). The higher similarity of 79.1% inferred between the Solheimajokull and Thingvellir populations may have been somewhat exaggerated by the large number of missing values substituting for floral characters in the Thingvellir population. On the other hand, these two populations both tend to have shorter stems, leaves and bracts, while Solheimajokull shares with Selfoss a tendency for longer bract cells and to develop one additional expanded leaf at the expense of subtracting one bracteoidal leaf. With regard to trends distinguishing single populations, Thingvellir had on average more flowers, Solheimajokull had wider, more spreading lateral sepals, Selfoss had larger spurs, and Stong had wider bracts but narrower stems and labella, and shorter ovaries (Table 2).

**Figure 10:**
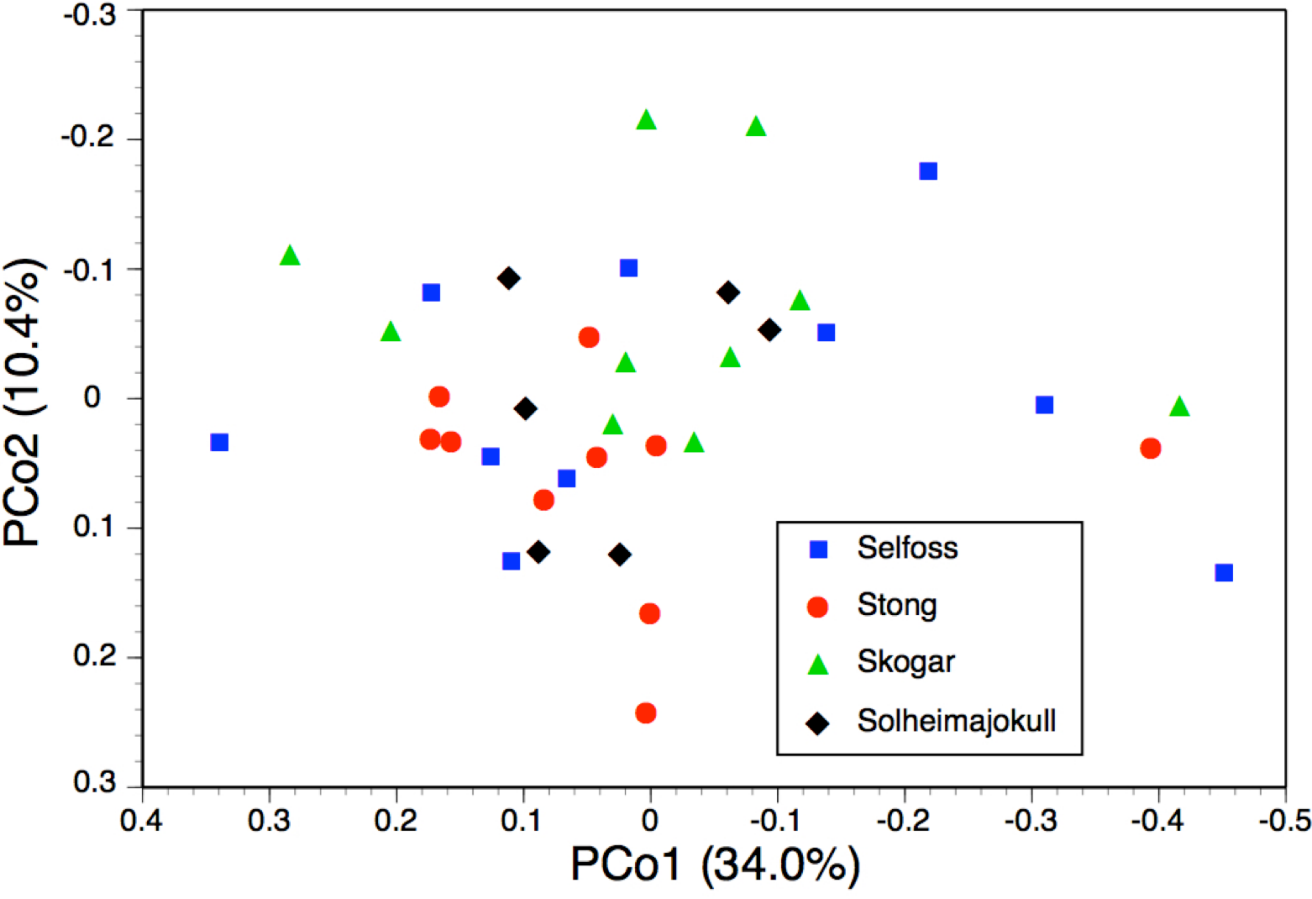
Bivariate scatter-diagram of the first two principal coordinates, derived from 28 morphometric characters measured for individual plants sampled in five Icelandic populations of *Platanthera hyperborea*. Parenthetic figures indicate the percentage of the total variance accounted for by each axis.

Once again, the PCo for individuals of *P. hyperborea* (Fig. 10) was much less discriminatory than the corresponding dendrogram. The comparatively strong first coordinate is effectively a size axis, plants toward the right-hand side of the plot having larger labella and other perianth segments, wider spurs, longer ovaries and basal bracts, together with longer inflorescences and wider stems. This axis offered little help in distinguishing between the study populations, though Selfoss evidently had the greatest proportion of large plants and Solheimajokull showed the lowest overall amount of size variation. The much weaker second coordinate gave partial separation of the Skogar and Stong populations, primarily on the basis of the more spreading lateral sepals of Skogar plants but also placing the more vegetively vigorous plants of all populations toward the upper end of the axis (Fig. 10). The yet weaker third axis (7.7%, not shown) served mainly to distinguish a single plant from Stong that (probably by happenstance) was the only individual measured to have either a slightly recurved labellum or bract marginal cells that were only moderately rather than strongly angular.

### Morphology: bivariate analyses

Table 2 gives mean, sample standard deviation and coefficient of variation values for all 38 morphometric characters measured in each of the five Icelandic populations of *P. hyperborea* measured by us.

**Figure 11:**
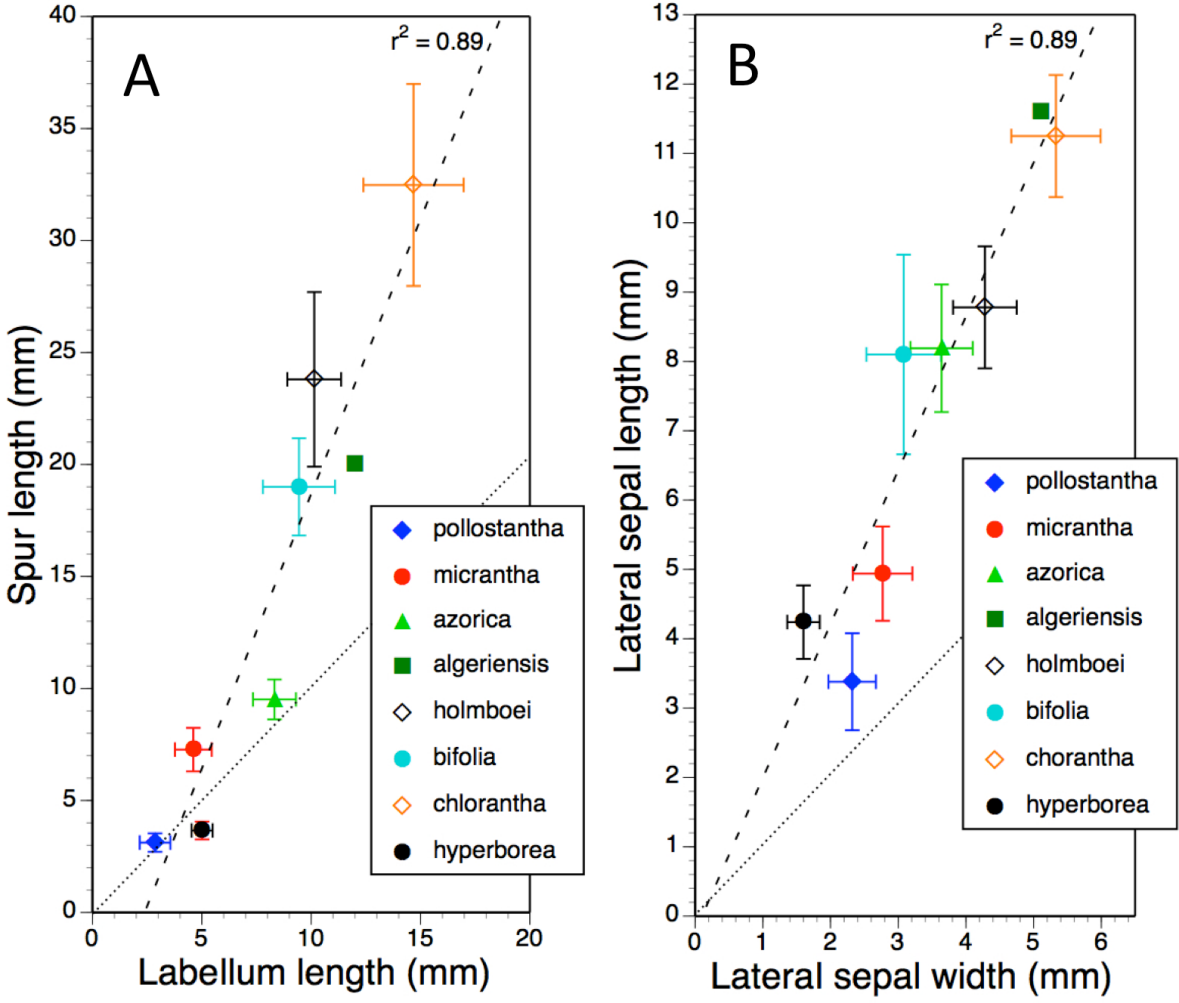
Bivariate plot of taxon mean values for (A) labellum length versus spur length and (B) lateral sepal width versus lateral sepal length for *Platanthera hyperborea* plus seven putative species and two hybrid combinations in *Platanthera* section *Platanthera*. Error bars represent sample standard deviations. The dashed line shows the regression, whereas the dotted line indicates parity in values of the two variables.

**Figure 12:**
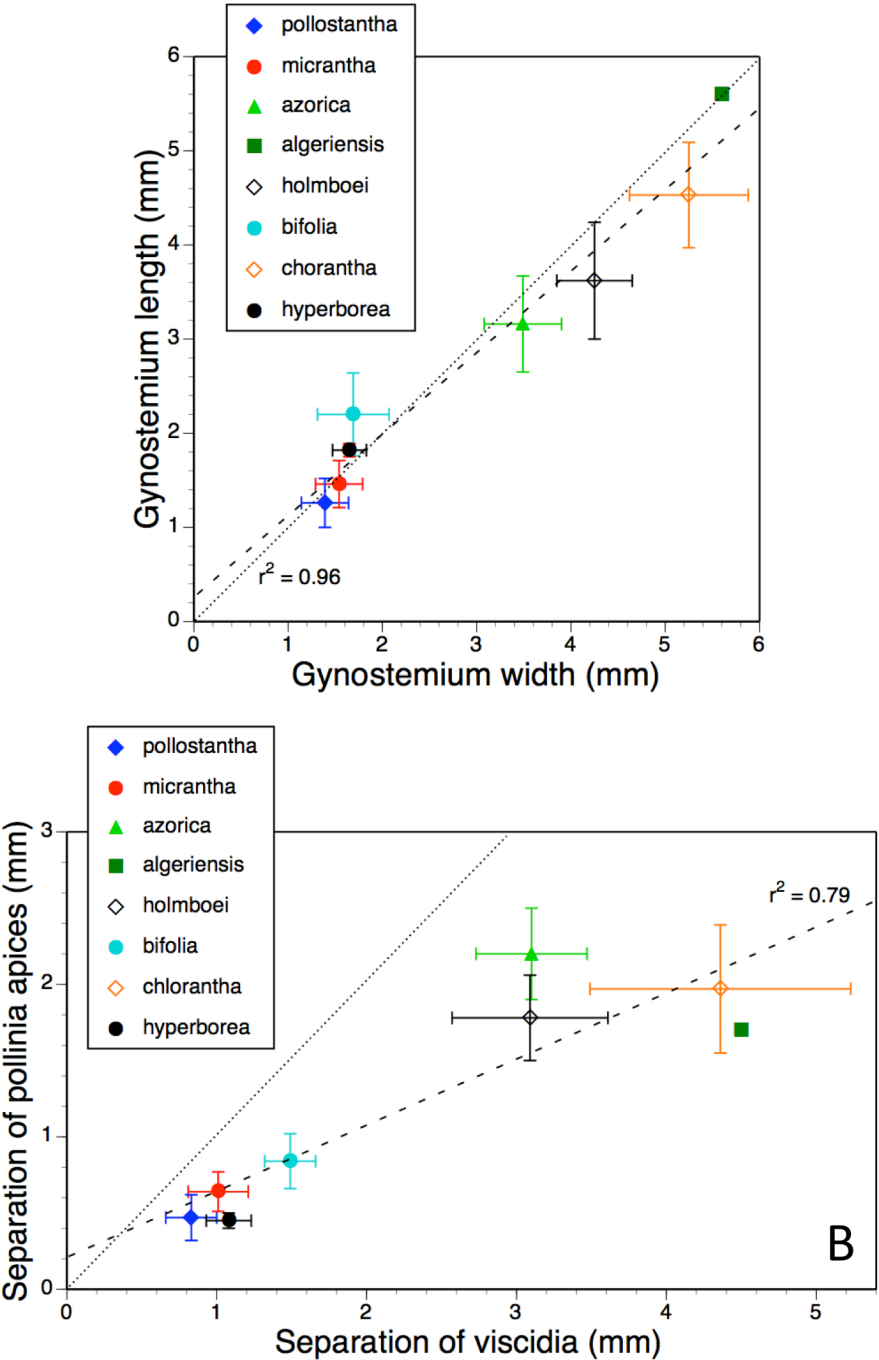
Bivariate plot of taxon mean values for (A) gynostemium width versus gynostemium length and (B) distance separating viscidia versus distance separating pollinarium apices for *Platanthera hyperborea* plus seven putative species and two hybrid combinations in *Platanthera* section *Platanthera*. Error bars represent sample standard deviations. The dashed line shows the regression, whereas the dotted line indicates parity in values of the two variables (i.e., parallel pollinaria in the case of B).

Mean values and standard deviations for metric characters of particular interest in all eight measured taxa were plotted together in pairwise combinations to yield scatter-diagrams that were subsequently subjected to regression. Ideally, type II regression would have been used. However, when *R*^2^ values are high (as here), reciprocal type I regressions (switching dependent and independent variables) reliably yields near-identical results. Examples of bivariate plots presented here are labellum length versus spur length (Fig. 11A), length versus width of lateral sepals (Fig. 11B), overall width versus length of gynostemium (Fig. 12A), and separation of the proximal ends of the pollinaria (i.e., the viscidia) versus separation of their distal apices (Fig. 12B). Each of these four plots provides substantial discrimination between most of the eight putative species of *Platanthera*. Although coefficients of variation are comparatively high (15–25%) for these eight metric parameters in most taxa, they are noticeably lower (<12%) for *P. azorica* and *P. hyperborea*. This difference can readily be explained for *P. azorica*, which effectively constitutes a single metapopulation (and thus represents a single gene pool occupying a comparatively narrow environmental spectrum), but it less clear why metric floral characters would be less variable in *P. hyperborea*.

The plot comparing labellum and spur lengths (Fig. 11A) shows a near-linear arrangement of mean values that generates a strong correlation (*R*^2^ = 0.89). Of the eight taxa, only *P. hyperborea* has a spur that is typically shorter than the blade of the labellum, matching the Azorean *P. pollostantha* in averaging a 3 mm spur but having a 5 mm rather than a 3 mm labellum.

Plotting mean values for lateral sepal width versus length (Fig. 11B) yielded an identical *R*^2^ value. The most deviant species is *P. pollostantha*, which has lateral sepals that are broader relative to their length than those of the other especially small-flowered species, *P. hyperborea*.

The linear relationship between the overall width and length of the gynostemium (Fig. 12A) is even stronger (*R*^2^ = 0.96), and in contrast with the other three plots, almost precisely tracks the dotted ‘line of parity’ between the two measures. The most deviant species is the comparatively long, narrow gynostemium of *P. bifolia*. The two small-flowered Azorean species, *P. pollostantha* and *P. micrantha*, both have gynostemia that are slightly smaller and more variable in size than those of *P. hyperborea*.

The eight species form two distinct clusters, four large and four small, on the plot comparing the basal and apical separation of the paired pollinaria (Fig. 12B). Variation within each of the clusters is sufficient to reduce the strength of the positive correlation (*R*^2^ = 0.79). Interestingly, none of the species achieves parity in the two measures. Although the pollinaria of *P. bifolia* are often described as parallel, in fact they fall precisely on the regression line that shows apical convergence in all measured taxa. *Platanthera hyperborea* equals *P. pollostantha* in having the most closely spaced apices, but has a slightly greater separation of the viscidia matching that of the other small-flowered Azorean species, *P. micrantha*.

### Morphology: synthesis

Typical plants of *P. hyperborea* are illustrated in Fig. 2. In vegetative architecture, *P. hyperborea* and other members of Section *Limnorchis* more closely resemble Orchidinae genera such as *Dactylorhiza* and *Gymnadenia* than Eurasian members of *Platanthera* section *Platanthera*. This is because *P. hyperborea* has several strongly keeled, lanceolate leaves that are borne in a spiral and decrease progressively in size up the robust stem until they grade into robust flower-subtending bracts, which similarly decrease in size toward the apex of the inflorescence. In contrast, species of Section *Platanthera* show a radical shift near the base of the stem from one or two weakly keeled, oval basal leaves to 3–4 lanceolate bracteoidal leaves (Bateman, Rudall & Moura, 2013; see also Webb, 1980; Sheviak, 2002). *Platanthera hyperborea* typically has three or four sheathing leaves, angled at *ca* 45° to the stem, 5–6 times as long as broad and typically widest at a point 35–40% of the distance outward from the stem; they grade into two or three non-sheathing bracteoidal leaves (Fig. 2B, Table 2). The bracts are robust and large, partially obscuring the lower flowers, and have a microscopically serrated margin. The relatively dense inflorescence is cylindrical and narrow, reflecting the acute angle subtended by the stem and ovaries; it typically constitutes 25–35% of the total stem length and contains 15–25 flowers.

When viewed in natural light, the flowers are uniformly translucent and yellowish-green in colour (145C–D or 149C–D on the RHS colour chart: Anonymous, 1966) (Fig. 2), but in typical flash images they appear opaque and somewhat yellower in hue (150A–C or 154A–B: Fig. 3); RHS colour blocks for both lighting regimes agree on a reflectivity value of 65–75%. The diversity of paler green and translucent white flowers evident in North American members of the *dilatata-hyperborea* group is thought to reflect differences in the density of chloroplasts in the floral organs (Bateman, Rudall & Moura, 2013). The oval labellum is typically 4.5–5.5 mm long and 1.5– 2.0 mm at its widest point, bearing a robust cylindrical spur that averages 3.5–4.0 mm in length and is curved strongly forward; the apical 30–50% contains nectar. The sepals are a little smaller than the labellum and the lateral sepals are a little smaller than the sepals. The dorsal sepal and lateral petals project forward to form a cowl-like hood over the compact but similarly forward-projecting gynostemium. In contrast, the lateral sepals and labellum are more spreading, though the precise postures of both categories of organ are dynamic, changing considerably following anthesis. The lateral sepals rapidly spread widely, becoming oriented closer to the vertical than the horizontal, but they then undergo gradual resupination at the base. The ensuing torsion within the lateral sepal means that, for the apical portion of the lateral sepal, it is the abaxial surface that faces forward rather than the adaxial surface. In some plants this resupination occurs rapidly after anthesis (Figs. 2C and 3B), whereas in other plants it is more gradual (Fig. 3A). Similar variation is evident in the posture of the labellum. In most plants the labellum rapidly progresses from a horizontal position to one that is near-vertical, but in a minority of individuals the labellum does not fully unfurl, remaining closer to the horizontal than the vertical and thus constraining access of insects to the spur and gynostemium.

**Figure 13:**
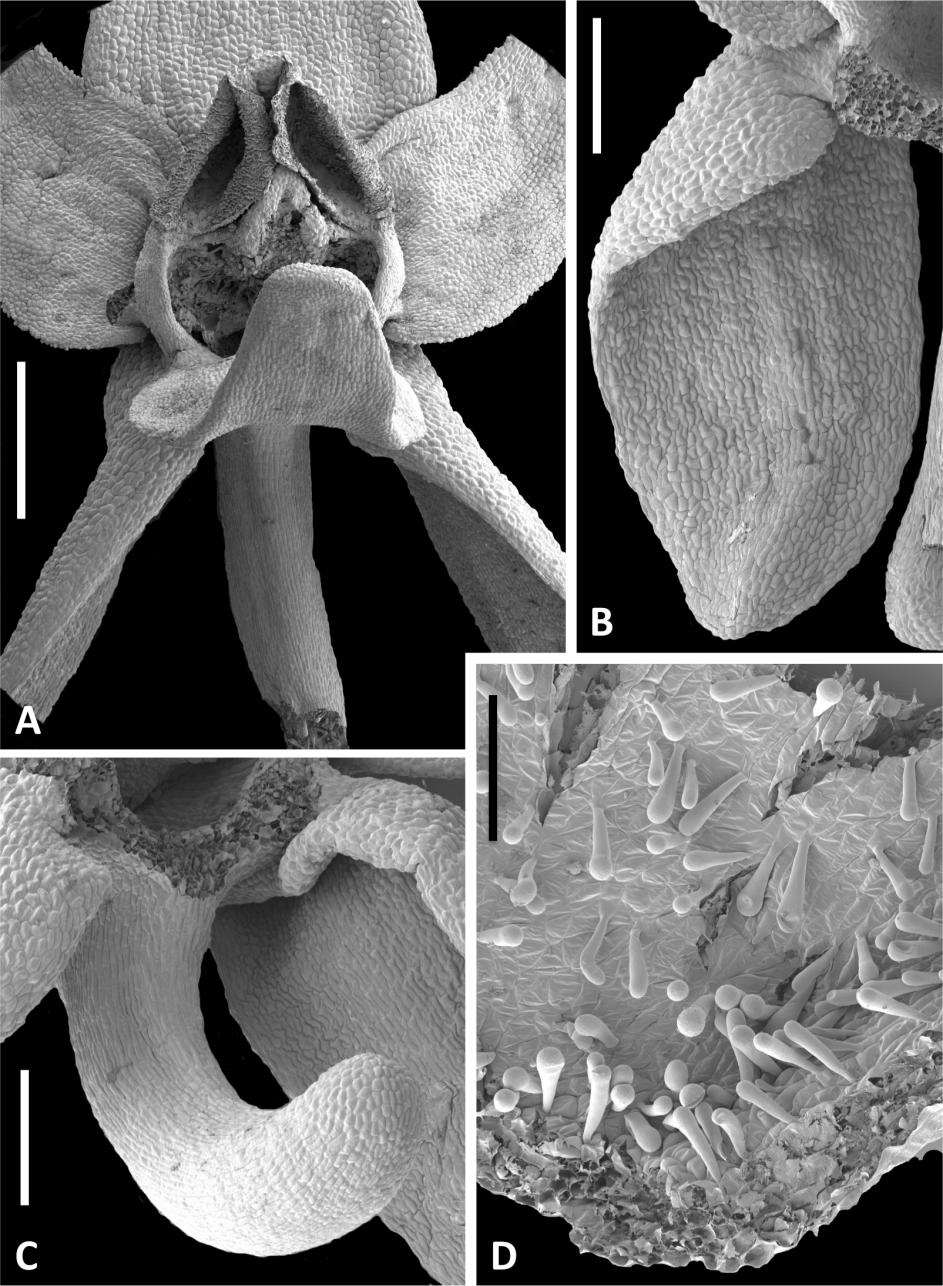
Scanning electron micrographs of flowers of Icelandic *P. hyperborea*. (A) Intact flower showing forward-projecting labellum, partially obscured ellipsoid spur entrance and triangular gynostemium. (B) Detail of left lateral sepal in a flower imaged later in anthesis; basal resupination has made visible the stomata-adorned adaxial surface. (C) Labellum removed to show the strong forward curvature of the spur and related longitudinally elongated epidermal cells. (D) Magnified image of cells lining the interior of the labellar spur apex, highlighting the distinctive club-shaped glandular papillae. Scale bar = 1 mm (A), 500 µm (B, C), 200 µm (D). Images: P Rudall.

**Figure 14:**
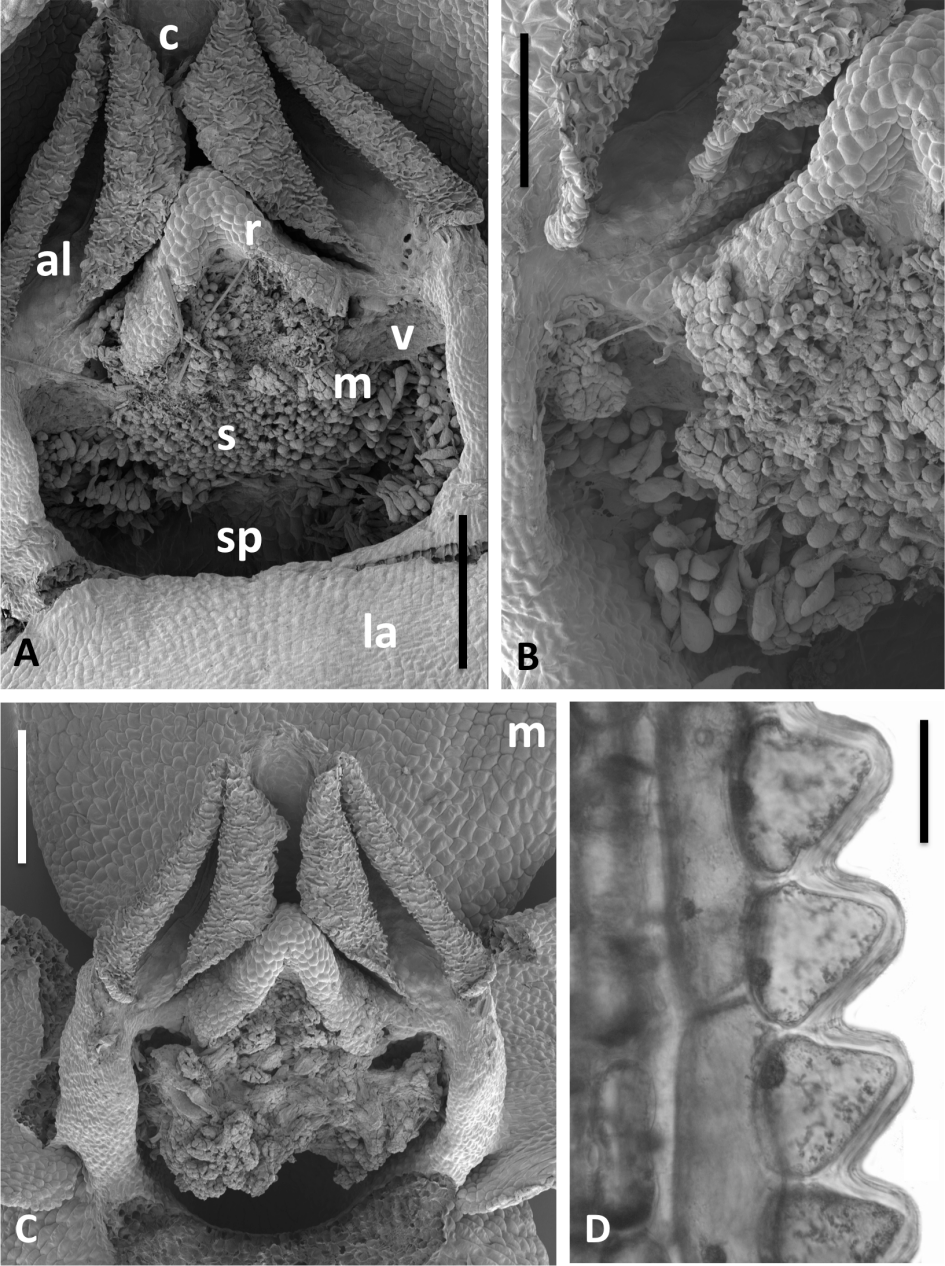
Scanning electron micrographs of flowers and bracts of Icelandic *P. hyperborea*. (A) Gymnostemium of recently opened flower, showing densely papillate stigmatic surface (s) bearing massulae (m) derived from the pollinaria that formerly occupied the now severely desiccated anther locules (al) bracketing the connective (c) and rostellum (r); also visible are depressions presumably formerly occupied by the viscidia (v), and the labellum (la). (B) Magnified view of (A) to better illustrate the stigmatic features. (C) Gynostemium of another flower that better illustrates the firm attachment of disaggregated pollinium fragments to the adhesive disc secreted by the stigma. (D) Distinctive row of robust, angular cells that characterises the bract margins of this species. Scale bar = 500 µm (A, C), 250 µm (B), 50 µm (D). Images: P Rudall.

**Figure 15:**
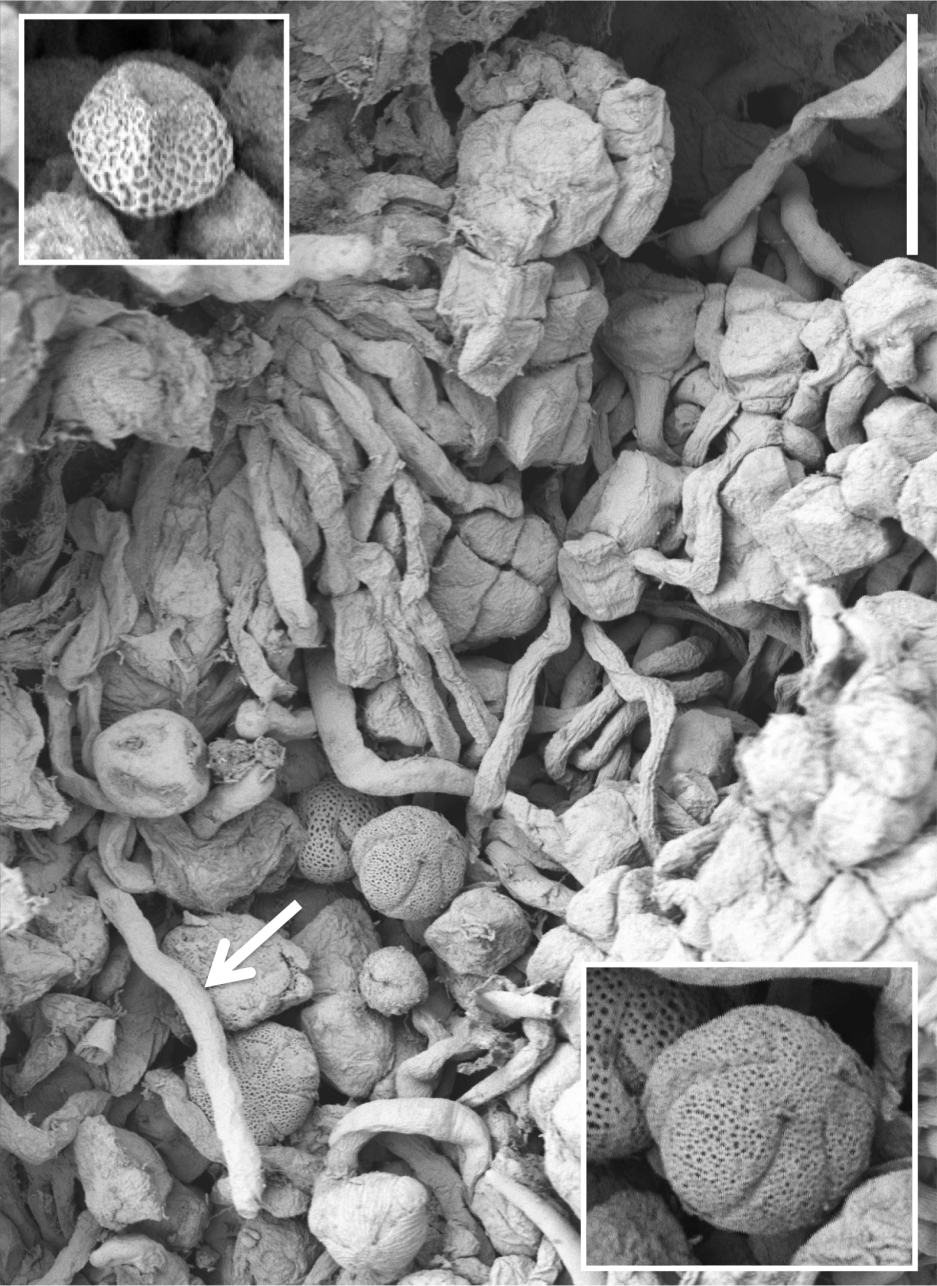
Scanning electron micrograph of numerous pollen grains adhering to the stigma of a *P. hyperborea* flower. The adjacent pollinium has disaggregated into massulae or smaller tetragonal tetrads that have germinated to generate a mass of pollen tubes (e.g., arrow). Insets show a triradiate spore suspected to have been derived from a nearby plant of the clubmoss *Huperzia selago* (top left) and a tricolpate pollen grain suspected to have been derived from a nearby plant of a labiate—most likely *Prunella vulgaris* but possibly *Thymus praecox* (bottom right). Scale bar of main image = 50 µm. Images: P Rudall.

The wide range of gynostemium morphologies exhibited by the genus *Platanthera s.l.* was surveyed by Efimov (2011). Within Section *Platanthera*, some details of *P. bifolia*, *P. chlorantha* and *P. holmboei* were illustrated by Claessens & Kleynen (2011) and Bateman, James & Rudall (2012), and later were compared with the three Azorean species plus *P. algeriensis* by Bateman, Rudall & Moura (2013). In order to adequately describe the gynostemium of *P. hyperborea*, and to obtain further details of the other floral structures, it proved necessary to examine representative flowers microscopically in the laboratory. Bracts were examined under the light microscope, demonstrating that the finely serrated margin reflects a distinctive row of comparatively large (80–90 µm) and thick-walled triangular cells (Fig. 14D). However, the small size, uniform colouration and translucent texture of the flowers of *P. hyperborea* meant that details of the floral parts were much more readily discerned through scanning than light microscopy. Flowers from five plants were examined and measured (Figs. 13–15, Table 2).

The flower illustrated in Fig. 13A has been manipulated to improve visibility of all organs; the median sepal and lateral petals have been swept backward to reveal the gynostemium, and the labellum has been oriented forward to reveal the spur. The torsion in the lateral sepals and decurvation of the spur are also discernible in Fig. 13A, though they are better seen in Figs. 13B and 13C, respectively. The abaxial surface of the sepal is evidently composed of smaller epidermal cells than the adaxial surface and bears scattered stomata—features that are absent from the adaxial surfaces of all six perianth segments. The spur describes a forward arc so well-developed that it approaches a semi-circle. Cells of its exterior epidermis are highly elongated except at the domed apex, suggesting that it probably elongates during anthesis (cf. Bateman & Sexton, 2008; Bateman, James & Rudall, 2012). Dissection of a spur revealed a wall approximately five cells thick and an internal epidermis that was evenly scattered throughout its length with unusual and distinctive club-shaped papillae (Fig. 13D).

Examination of several gynostemia revealed that these papillae extend upward from the broad spur entrance to the stigmatic surface, where they seem more densely packed (Figs. 14A and 14B). The stigma appears to be relatively broad but shallow and unipartite, though this is difficult to demonstrate conclusively, as the stigma is reliably partly (Figs. 14A and 14B) or wholly (Fig. 14C) enveloped by a thick glue-like plug that is liberally decorated with massulae—the crumbled remnant of the formerly suprajacent pollinaria (Fig. 15). Also frequently attached to this plug are spores and pollen grains of various co-habitating plant species (insets in Fig. 15), together with fragments of insects, most notably mosquitoes. Massed pollen tubes are reliably evident emerging from isolated or still aggregated permanent tetrads (Fig. 15).

We are unable to describe the pollinaria, as even in unopened buds, as they had already disaggregated onto the more proximal regions of the gynostemium. Their overall length can be calculated from the empty thecae, and appropriately placed depressions at the base of the thecae suggest that the viscidia were near-circular in outline (mirroring the single viscidium of *P. convallariifolia* illustrated in Fig. 2D of Efimov, 2011, though the viscidia of *P. hyperborea* were described as “linear to linear-oblong” by Sheviak, 2002) and as presented were inclined only slightly relative to the horizontal (Figs. 13A, 14A–14C). We infer the caudicles to have been exceptionally short.

The locular apertures are linear, in contrast with the sigmoid apertures of the larger-flowered of the species within Section *Platanthera*. In *P. hyperborea* the thecal walls appear exceptionally desiccated even prior to anthesis, the cells of the interior epidermis resembling a layer of cornflakes or fish-scales (Figs. 14A and 14C). The pair of presumed viscidia are comparatively widely separated and, as in most species of *Platanthera*, the thecae converge distally toward the narrow connective. The rostellum is compact and angular, and so is easily laterally by-passed by falling massulae released as the pollinia fragment. Apart from the stigmatic surface, the flowers of *P. hyperborea* are remarkably free of cellular adornments.

Lastly, although we did not examine the seeds of *P. hyperborea*, previous SEM-based studies of the seeds of ‘*P. hyperborea*’ (Healey, Michaud & Arditti, 1980; probably actually *P. huronensis*) and of *P. dilatata* plus *P. aquilonis* (Gamarra et al., 2008) reported the testa as being clavate with a rounded apex, and consisting of cells of a near-uniform size that have smooth periclinal walls and lamellate anticlinal walls.

### Formal description

All dimensions refer to fresh rather than dried plants in order to obtain measurements that are both reliable and relevant to field biologists (cf. Bateman, Rudall & Moura, 2013; Parnell et al., 2013). Variance in metric and meristic characters is given to two standard deviations, thereby in theory encompassing 96% of the plants measured. Variance in scalar characters is indicated by the following terms: usually = >80%, often = 51–80%, occasionally = 20–50%, rarely = <20%.

**Iceland Butterfly-orchid** (= Northern Butterfly-orchid, Northern Green-orchid)

*Platanthera hyperborea* (L.) Lindl., Gen. Sp. Orchid. Pl. 287. 1835

Basionym: *Orchis hyperborea* L., Mant. Pl. 1: 121. 1767

Synonyms: *Habenaria hyperborea* (L.) R.Br. in W.T. Aiton, Hort. Kew. (ed. 2) 5: 193. 1813

*Gymnadenia hyperborea* (L.) Link, Handbuch [Link] i: 242. 1829

*Limnorchis hyperborea* (L.) Rydb., Mem. N. Y. Bot. Gard. 1: 104. 1900

Tubers narrowly fusiform to filiform, tapering to a single, long, fleshy apical root; a few further roots emerge horizontally from the base of the stem. Stem 16 ± 11 cm, 3.4 ± 2.3 mm in diameter. Sheathing leaves 3.3 ± 1.5, largest 71 ± 32 mm × 13 ± 7 mm, broadly lanceolate (widest 30–40% of the distance from the base), usually keeled, acutely angled and lacking a distinct petiole; bracteoidal leaves 2.1 ± 1.5, usually distributed fairly evenly along stem and grading into basal bracts. Inflorescence 45 ± 37 mm, 18 ± 18 flowers (*ca* 4.0 fls/cm). Basal bracts 14 ± 7 mm, floral bracts 9.4 ± 2.4 mm × 2.4 ± 0.5 mm, lanceolate; marginal cells thick-walled and strongly triangular, 85 ± 24 µm in longitudinal diameter. Flowers uniformly pea green to sap green/chartreuse green (RHS 145C–D or 149C–D in natural light, 150A–C or 154A–B in artificial flash), somewhat translucent; median sepal and lateral petals connivent over gynostemium. Labellum entire, 5.0 ± 1.0 × 1.8 ± 0.5 mm, elliptic-ovate, held vertically or more often projecting slightly forward, occasionally also curved gently upward. Spur 3.7 ± 0.8 mm long × 0.8 ± 0.3 mm in diameter at mouth, 0.9 ± 0.2 mm midway along its length, cylindrical, strongly down-curved, containing club-shaped papillae throughout; spur entrance moderately compressed vertically; apical-most 25–50% filled with nectar. Ovary 9.1 ± 2.4 mm. Lateral sepals oriented closer to vertical than horizontal, 4.2 ± 1.1 × 1.6 ± 0.5 mm, becoming resupinate in mature flowers. Lateral petals 3.4 ± 0.8 mm. Gynostemium 1.8 ± 0.2 mm long × 1.7 ± 0.4 mm wide; stigma grades into spur entrance, a horizontally elongate oblong, at most 1.2 ± 0.3 mm wide, stigmatic surface densely covered in club-shaped papillae that secrete a white, translucent, glutinous plug; rostellum compact, a subdued ∧-shaped ledge; auricles absent. Anther locules linked by a narrow, comparatively thin connective, locule aperture ± linear, extremely relaxed, inner cells desiccated and resembling fish-scales; paired pollinaria *ca* 1.0 mm, moderately convergent from viscidium to pollinium apex, viscidia separated by 1.1 ± 0.3 mm, apices of pollinaria separated by 0.5 ± 0.1 mm; viscidia pendent, angled downward and slightly forward, not opposed, near-equidimensional; caudicle near-linear, strap-like, much shorter than the protruding, pale yellow pollinium. Fragrance absent or at most weak. 2*n* = 42. ITS1 includes the motif T**G**TCCTCAAAACGAAA**T**GA (rarely with the alternative ending **C**GA).

*Distribution:* Given the morphological and molecular ambiguities within this species complex, *P. hyperborea* can only be considered with certainty to occur on Iceland. However, it seems likely from ITS data that the species also extends through Greenland and Arctic Canada at least as far west as Alaska.

*Habitat:* A speclialist in tundra and taiga habitats. Sun and occasionally semi-shade in moist acidic soils (occasionally ever-wet flushes); typically occurs in boggy heathland or river floodplains, but also along the margins of open scrubby woodlands (often *Salix*-dominated) and in sparse grasslands on drier soils; from sea level upward (its close relatives occasionally reach at least 2000 m asl).

*Flowering:* (Early June–)mid-June–mid-July(–late July).

*Holotype:* Linnean 1054.42, “Island” [= Iceland]. Reputedly “Oxeraa, Iceland, 1767” (presumably the River Oxeraa, most likely close to Thingvellir in southwest Iceland).

*Present illustrations:* Figs. 2, 3 and 13–15.

*Etymology:* The Linnean epithet derived from the Greek *hyper-* (beyond, over, above) and *boreas* (north [wind]), reflecting the apparently semi-circumboreal distribution of this classic tundra species. The colloquial name Iceland Butterfly-orchid has been devised here to replace the ambiguity inherent in previous names used for this orchid: Northern Butterfly-orchid and Green-flowered Butterfly-orchid.

### Comparison with previous morphological descriptions of *P. hyperborea*

When assessing the accuracy of previous descriptions of *P. hyperborea* relative to our morphometric matrix (Table 2), it is difficult to separate the effects of authors adopting a broader circumscription of *P. hyperborea* by including North American segregates (most notably *P. aquilonis* and/or *P. huronensis*) from the many other potential sources of error. This phenomenon probably accounts for descriptions that give 2*n* = 42 + 84 or, more often, simply as 2*n* = 84 (e.g., Löve & Ritchie, 1966; Sundermann, 1980; Webb, 1980; Sheviak, 2002), when the only chromosome count based on Icelandic material that we have found in the literature is actually 2*n* = 42 (given by Dalgaard, 1989). The peak flowering period is often given as July–August (e.g., Buttler, 1991; Sheviak, 2002; Delforge, 2006), whereas in all of our study populations flowering peaked during a surprisingly narrow interval between the third week of June and first week of July in 2014 (Table 1; admittedly, they all occurred at low altitudes, below 160 m asl). Where metric measurements have been given for flowers they tended to exaggerate the size of the labellum, spur and/or lateral sepals (Webb, 1980; Baumann & Künkele, 1988; Davies, Davies & Huxley, 1983; Buttler, 1991; Sheviak, 2002; Delforge, 2006; Claessens & Kleynen, 2011), whereas the treatment by Luer (1975) greatly exaggerated the upper boundaries for several vegetative characters compared with Icelandic populations.

### Comparison with previous morphological descriptions of Section *Platanthera*

Vegetatively, *P. hyperborea* and its close relatives differ from Section *Platanthera* in having narrower tubers (e.g., Sundermann, 1980; Currah, Smreciu & Hambleton, 1990; Sheviak, 2002; Sheviak & Jennings, 2006; Colwell, Sheviak & Moore, 2007; Efimov, 2011) that arguably are better described as filiform than fusiform. Rather than showing a clear distinction between oval basal leaves and lanceolate bracteoidal leaves, those of the *P. hyperborea* group pass gradually from broadly lanceolate lower leaves to bracteoidal upper leaves. The most obvious difference in the perianths of the two Sections is the post-anthesis resupination that affects the lateral sepals of *P. hyperborea*.

But the majority of the differences between the Sections are smaller in scale. The serrate margins of the bracts of *P. hyperborea* contrast with the smooth margins of Section *Platanthera*, and although *P. hyperborea* shares with the larger-flowered species in Section *Platanthera* the presence of papillae within the spur, they are clavate rather than parallel-sided and spread across the stigma. Unlike species of Section *Platanthera*, we were unable to detect stomata on the adaxial surfaces of any of the perianth segments in *P. hyperborea*, or to find any evidence of potential osmophores (scent-secreting cells) on the labellum. Lastly, the botryoidal auricles that reliably bracket the gynostemium in all species of Section *Platanthera*—prominent in large-flowered species, more subdued in small-flowered species—appear to be wholly absent from *P. hyperborea* (Efimov, 2011, argued that they were present in members of Section *Limnorchis* but very small).

### Broader implications for morphometric approaches to phylogeny reconstruction and species delimitation

As recommended by Bateman (2001) and Bateman (2012), the present study presents multivariate analyses conducted at three contrasting demographic levels: individuals (Figs. 7 and 10), populations (Fig. 9), and species (Fig. 8). The present results make an interesting comparison between these levels.

The two analyses confined to *P. hyperborea s.s.* are consistent and complementary. The principal coordinates plot of individual plants within these populations (Fig. 10) showed almost complete overlap of the four populations, and revealed similar levels of variance in three (variance appears to be somewhat lower in the Solheimajokull population). This overlap was easily explained once it became clear that the strong first axis largely represents the sizes of various organs (several floral and vegetative features in PCo1, several size parameters plus lateral sepal position in PCo2), which increase toward the top right of the plot. Thus, most of the variance that we detected simply reflects plant size, and at Solheimajokull we found neither exceptionally small nor exceptionally large plants. Although the size of the plants is likely to include a genetic component, it largely reflects the vigour of the plant during that particular year, which in terrestrial orchids is dominantly influenced by non-genetic ecophenotypic factors and, in the case of juvenile plants, by age since germination (Bateman & Denholm, 1989). When the character data were summarised as population means, the resulting dendrogram (Fig. 9) unsurprisingly showed the populations to have approximately equal similarities with each other.

Moving on to the ordination of individuals that compares *P. hyperborea* with seven members of Section *Platanthera* (Fig. 7), it is clear that *P. hyperborea* is potentially a distinct species, but there is no substantial morphological discontinuity to suggest that it represents a different taxonomic section. Indeed, the scores for plants of *P. hyperborea* are identical to those of the Azorean *P. micrantha* on PCo1 and identical to those of the European *P. bifolia* on the substantially weaker PCo2. However, a contrasting picture emerges once these raw data have been summarised as taxon mean values. The resulting dendrogram (Fig. 8) clearly shows the greater morphological disparity between *P. hyperborea* and members of Section *Platanthera* compared with disparities among species within the Section—though admittedly the disparity is enhanced by the addition to the matrix of two characters representing the presence of clavate papillae on the spur and stigma.

The main reason for the difference between the ordination based on data for individuals and the dendrogram based on taxon means is that the taxon-level analyses average out the effects of variation in the sizes of various organs brought about by contrasts in plant vigour; these dominate the first axis of the ordinations based on individuals (Figs. 7 and 10). Thus, for morphometric data, individual-level and population-level analyses are most valuable for circumscribing taxa (e.g., Bateman, 2001; Bateman, 2012), whereas taxon-level analyses give a more accurate representation of the relationships among taxa once they have been circumscribed with sufficient confidence.

The effects of ecophenotypy on a morphometric matrix are greatest when it consists entirely of continuous metric characters, such that one highly influential emergent property (the vigour of the plant in question) can dictate values for every character included in the matrix. Unfortunately, all of the morphometric matrices gathered on North American members of the *P. dilatata-hyperborea* group have consisted entirely of a compartively small number of continuous metric characters employed to describe floral parts. Nonetheless, it proved possible (just) to use these characters to discriminate between *P. dilatata*, *P. aquilonis* and their F1 hybrids or their allotetraploid product *P. huronenesis* (Catling & Catling, 1997; Wallace, 2006; Sears, 2008), and between two of the three varieties of *P. dilatata* (Adhikari & Wallace, 2014).

### Is biologically meaningful structure present within Icelandic *P. hyperborea*?

Fortunately, there has been general agreement that, whereas there is considerable taxonomic complexity within Section *Limnorchis* in North America, only *P. hyperborea s.s*. occurs on Iceland (e.g., Delforge, 2006). Nonetheless, Sheviak (2002, p. 559) stated that “In both [Greenland and Iceland], considerable variation occurs, and some plants suggest *P. huronensis*. Whether this variation reflects the occurrence in these two areas of two taxa or is within *P. hyperborea* is unknown.”

The possibility that the eastern North American lineage of the allotetraploid *P. huronensis* extends north-eastward from the margin of its accepted range in Newfoundland and Labrador is an interesting one. However, we found morphological variation among our five study populations of *P. hyperborea* (Figs. 9 and 10) to be surprisingly low compared with other north-temperate orchid groups studied by us using similar morphometric approaches, not least the *P. bifolia-chlorantha* group (Bateman, James & Rudall, 2012; Bateman, Rudall & Moura, 2013; Bateman et al., 2014). This phenotypic consistency extends to the size of the bract marginal cells, which has proven to be a useful indicator of ploidy level in the closely related genus *Dactylorhiza*. Specifically, mean cell size varied only from 80 µm at Skogar to 91 µm at Selfoss (Table 2); such differences are too small to be likely to indicate the presence of multiple ploidy levels. The contrasting ITS ribotype of the Solheimajokull population relative to the remainder (Fig. 6) could potentially indicate taxonomic structure at the genotypic level, but there is no suggestion of any corresponding divergence in the phenotype (Figs. 9 and 10) or phenology of this population.

However, our pragmatic decision to sample only low-altitude (below 200 m asl: Table 1) populations of *P. hyperborea* leaves open a small possibility that greater genotypic and phenotypic diversity could conceivably exist at higher altitudes. Most of Iceland above 1100 m asl is presently covered by permanent ice, and much of the remainder is officially categorised as unvegetated, the (largely nominal) treeline currently approximating 250 m asl. Nonetheless, there remains a considerable altitudinal range for the orchid to potentially inhabit—one that could aid the species’ future well-being if the regional climate continues to warm, driving upward the communities preferred by *P. hyperborea* (cf. Colwell & Lees, 2000).

### Remarkable reproductive biology of *P. hyperborea*

Members of the genus *Platanthera* have underpinned some of the most detailed studies of pollination biology in the orchid family. Thus far, the emphasis has been placed on adaptations for insect-mediated allogamy, most commonly involving moths (e.g., Darwin, 1877; Nilsson, 1983; Maad & Nilsson, 2004; Little, Dieringer & Romano, 2005; Boberg et al., 2014). Indeed, the *P. dilatata-hyperborea* group encompasses multiple reproductive strategies, many members being pollinated primarily as a result of offering nectar rewards to various noctuid moths (Luer, 1975; Hapeman & Inoue, 1997; Sheviak, 2002). Strongly nectariferous orchid species generally attract wider spectra of pollinating insects than related species that practice various forms of deceit (e.g., Cozzolino & Widmer, 2005; Claessens & Kleynen, 2011). On the other hand, the range of potential pollinating insect species available to a temperate orchid declines precipitously as its distribution approaches the Arctic Circle; other reproductive strategies may then become more effective (e.g., Cheptou, 2012). This fact is not wholly surprising, as it has become conventional wisdom that the frequency of autogamy within floras increases toward the geographic poles as the spectra of potential pollinators declines (cf., Hagerup, 1952; Kevan, 1972; Strathdee & Bale, 1998). Orchids are no exception (reviewed by Catling, 1990).

Most of the comparatively few authors who have definitely been reporting observations of *P. hyperborea s.s.* simply describe its reproductive strategy as autogamy, though a few have offered greater detail. Delforge (2006, p. 145) described the spur as “slightly nectariferous,” the flowers as “sweetly scented,” and the plants as “facultatively self-pollinating or cleistogamous.” Our observations suggest that the spur is strongly nectariferous (although the spur is short, it is generally filled to 25–50% of its length) and the flowers emit little if any fragrance (our SEM studies failed to identify any obvious scent-secreting osmophores: Figs. 13 and 14). But we certainly agree that the flowers are routinely pollinated through cleistogamous autogamy.

At least 90% of *P. hyperborea* flowers set copious seed in both Iceland (our observations) and Greenland (Hagerup, 1952), a fact that generally provides the first piece of circumstantial evidence of either self-pollination or apomixis in a plant species. The reliable presence of innumerable pollen tubes entering the stigmatic surface (Fig. 15) of each flower clearly rules out both apomixis and any form of intrinsic sterility barrier. Our microscopic examination of *P. hyperborea* flowers failed to reveal any open flower, opening flower or (most convincingly) closed bud that lacked massulae attached to, and germinating on, the stigmatic plug (Fig. 15); cleistogamy is evidently routine in this species. Some previous authors (e.g., Gray, 1862; Reinhard, 1977; Catling, 1983; Catling, 1990; Delforge, 2006; Claessens & Kleynen, 2011) have implied that the mode of autogamy is that epitomised by the bee orchid, *Ophrys apifera*, whereby the caudicles are insufficiently robust to maintain within the locules erect pollinia, which consequently swing downward under the pull of gravity, contacting the stigma as single units. However, other authors simply referred to the presence on the stigma of disaggregated massulae (Hagerup, 1952; Catling, 1990; Sheviak, 2000; Claessens & Kleynen, 2011), presumably the result of crumbling of the pollinia onto the underlying stigmatic surface, passing over and/or down either side of the minimalistic rostellum (Fig. 14A–14C). We have gained circumstantial evidence that both processes operate in tandem in *P. hyperborea*. In either case, attachment of pollen masses to the stigma would likely be enhanced by the strong winds and driving rain that characterise the Icelandic climate.

We are confident that Hagerup (1952, p. 53) was correct in stating that the massulae typically reach the stigma prior to anthesis, having been released through early and rapid dehydration of the anther walls. The precocious timing and impressive intensity of that dehydration process is testified by the remarkable tessellated texture of the locule lining evident at the time of dehiscence of the anthers (Figs. 13A, 14A and 14C). A similar effect was reported by Bateman, Rudall & Moura (2013) in the locules of the small-flowered Azorean species *P. pollostantha* and *P. micrantha*. However, we hypothesise that in *P. hyperborea* the dehydration process extends to both the pollinaria and the stigmatic surface. In the case of the pollinaria, dehydration not only causes the pollinia to become more friable but also renders non-functional the caudicles and viscidia. On the stigmatic surface, withdrawal of moisture increases the viscidity of the initially more fluid stigmatic exudate (Figs. 14B and 14C). Thus, dehydration of the gynostemium drives both male and female functions toward autogamy.

We suspect that the distinctive clavate papillae observed in both the interior of the spur and the stigmatic surface by both Hagerup (1952) and ourselves (Figs. 13D, 14A and 14B) also play multiple roles in the reproductive biology of these flowers. Papillae adorning the stigma are likely to be responsible for secreting the unusually large and persistent stigmatic plug, whereas those within the spur fulfill their more traditional role of secreting (and perhaps later resorbing) significant volumes of nectar (Bell et al., 2009). Interestingly, although most members of Section *Platanthera* possess papillae within their spurs (albeit cylindrical in shape, and with distributions that never extend proximally to the stigmas), the papillae have been evolutionarily lost from the small-flowered Azorean endemics *P. pollostantha* and *P. micrantha*, which nonetheless continue to produce significant quantities of nectar (Bateman, Rudall & Moura, 2013).

However, these small-flowered Azorean species are suspected of remaining insect-pollinated (Bateman, Rudall & Moura, 2013), unlike the autogamous *P. hyperborea*. Indeed, conventional evolutionary wisdom would state that natural selection should have eliminated nectar production in this species, as it represents a substantial waste of energy if pollinator attraction is no longer a requirement to maintain populations. We speculate that continued secretion of nectar by the clavate papillae within the spur is the price that must be paid by the orchid if the identical clavate papillae located on the stigmatic surface are to continue to generate the large quantities of exudate needed to maximise capture of the crumbling massulae. In other words, we hypothesise that it is physiologically simplest to withdraw moisture from the entire distal portion of the gynostemium, affecting both the pollinaria and the stigma, and it is physiologically simplest to secrete sugar-rich exudates from all clavate papillae present on the flower, irrespective of whether they line the spur or adorn the stigma. If so, these characters represent classic examples of evolutionary trade-offs.

Several authors have speculated that *P. hyperborea* may be pollinated by mosquitoes. This suggestion receives some encouragement from the unequivocal demonstration that *Aedes* mosquitoes contribute substantially to North American populations of the similarly near-circumboreal butterfly-orchid species *Platanthera* (*Lysiella*) *obtusata* (Stoutamire, 1968; Thien, 1969; Hapeman & Inoue, 1997; Claessens & Kleynen, 2011), which generally attaches its pollinaria to their heads—though it later emerged that the primary pollinators of this species are more likely to be *Xanthorhoe* moths (Thien & Utech, 1970). Although female mosquitoes are best known for either requiring or at least benefiting from a blood meal prior to egg production, both females and males rely primarily on nectar and other monosaccharide-rich plant exudates for nutrition, most commonly obtaining them at night (e.g., Andersson & Jaenson, 1987).

We frequently found single moribund mosquitoes at least partially blocking access to the nectar spur and stigma of *P. hyperborea* (Fig. 3B); this was true of only a minority of the flowers in some populations, but in others, almost every open flower contained a mosquito—those within older flowers were often partially decayed. Their presence suggests that the substantial reservoirs of nectar in the modest-sized spurs of *P. hyperborea* constitute a valuable food source for at least some of the mosquito species that occur on Iceland. However, it is far less clear whether this symbiotic relationship brings any benefits to the orchid. The extreme curvature of not only the spur but also the overhanging stigma, and the tendency of the labellum to further obscure the entrance of recently opened flowers (Fig. 2C), almost certainly brings a high proportion of visiting insects into contact with the stigmatic surface. And although the stigmas of many *Platanthera* species have been shown to be coated in pale adhesive exudate (e.g., Bateman, Rudall & Moura, 2013), the stigma of *P. hyperborea* is remarkable for generating an exceptionally large and sticky plug—one that, by anthesis, resembles used chewing-gum in both appearance and texture. The plug is clearly efficient at capturing not only cascading massulae but also pollen grains of other plant families (Fig. 15, insets) and other plant debris. It seems likely that any mosquito that touches this plug will become fatally attached, thereby precluding transfer of pollen massulae from one flower to another. If so, the only contribution likely to be made by the insect to pollination of the orchid would be through dislodging massulae from the overhanging pollinia onto the subjacent stigma during its death-throes. The insects would then be contributing to autogamy, rather than fulfilling their more traditional role of achieving allogamy.

In summary, there are four possible explanations for the frequent occurrence of moquitoes within the flowers of *P. hyperborea*:

(1) They frequently pollinate the flowers through transfer of pollinaria between flowers, commonly of different plants, thereby acting as primary pollinators;
(2) They occasionally transfer massulae between plants, thereby maintaining a level of gene flow that is modest but nonetheless sufficient to preclude deep inbreeding depression;
(3) They aid self-pollination of the flowers as they expire through vibrations that further disaggregate the already desiccated pollinia residing above the stigma;
(4) They play no role in the pollination of the orchid, simply being unfortunate bystanders.

We suspect that the correct answer is either (3) or (4). In this context, it would now be helpful to gather population genetic data in order to estimate the strength of gene flow (if any) occurring among *P. hyperborea* populations (cf. Squirrell et al., 2002).

### Allometric and paedomorphic reduction in flower size in island *Platanthera*s

In previous papers (Bateman, Rudall & Moura, 2013; Bateman et al., 2014) we have explored in detail the likely origin(s) of *Platanthera pollostantha* and *P. micrantha*, the two species endemic to the Azorean archipelago that possess small green flowers. Speciation was achieved via reductions in the sizes of floral organs—some allometric, others heterochronic—from much larger-flowered ancestor(s) in Section *Platanthera*. In Icelandic *P. hyperborea*, we have again encountered a phylogenetically derived species that possesses small green flowers and occupies a mid-Atlantic island. And on the Hawaiian archipelago, in the centre of the Pacific Ocean, resides another island endemic (Torres-Santana, Bruegmann & Zablan, 2007) that appears to be derived from a somewhat larger-flowered member of Section *Limnorchis* (Bateman et al., 2014). Current evidence suggests that these speciation events represent three independent origins of lineages, two (Iceland, Hawaii) being derived from closely related ancestors, the third (Azores) from an ancestor only distantly related to the other two. This observation raises the question of whether comparing these three evolutionary transitions could allow more general conclusions regarding (a) the degree to which these transitions deviate from allometry between developmentally correlated features and (b) whether there is a minimum size below which a *Platanthera* flower becomes sufficiently dysfunctional to be evolutionarily inviable.

In this context, the regressions of floral dimensions (Figs. 11 and 12) are especially relevant. The two graphs representing perianth characters show equal and strong correlation coefficients (Fig. 11). Sepal dimensions unequivocally represent a functional constraint as the sepals enclose the petals and gynostemium during floral development; if the petals (including the labellum) are to develop in size beyond that of the sepals, they must do so following anthesis, either by unrolling or by continued growth (cf. Bateman & Sexton, 2008). The correlation line for these two parameters (Fig. 11B) passes through the origin of the graph and shows that width is typically 45% of length. In contrast, the regression line for length of the labellar blade versus the labellar spur (Fig. 11A) does not pass through the origin; spur length averages approximately twice the length of labellum length in the larger-flowered species but the two parameters approximate parity in the smaller flowered species. Moreover, *P. hyperborea* and *P. micrantha* have identical mean labellum lengths but the spur of *P. micrantha* averages twice the spur length of *P. hyperborea*.

When considering gynostemium dimensions (Fig. 12), the eight species cluster in two groups of four species each—one group large-flowered, the other small. In the case of the overall dimensons of the gynostemium (Fig. 12A), the correlation is remarkably strong and, moreover, shows equidimensionality between length and width. It is therefore tempting to view the gynostemium as operating under an unusually strong developmental constraint. It makes an intriguing contrast with the positioning of the pollinaria within the gynostemium, where the viscidia are typically separated by twice the distance between the pollinium apices (Fig. 12B). The strength of the correlation is lower than that of the other paired measures figured here, though nonetheless strong. First principles suggest that the separation of the pollinium apices has little relevance to pollination biology and is more likely to be constrained by the overall size and (remarkably consistent) shape of the gynostemium. In contrast, the distance separating the viscidia should in theory be under strong selection encouraging placement on the appropriate portion of the preferred pollinating insect(s). If so, deviation from the allometric norm should be greater in this parameter. However, this is not the case, nor is variation *within* the species in this parameter substantially less; coefficients of variation within the seven measurable species average 16% for viscidial separation and 19% for apical separation.

When seeking to identify size contraints on miniaturisation of *Platanthera* flowers, strong similarities are evident in the dimensions of Icelandic *P. hyperborea* and the smallest-flowered of the three Azorean species, *P. pollostantha* (Figs. 11 and 12). For all floral organ dimensions other than sepal width and separation of pollinium apices, *P. pollostantha* has achieved slightly smaller mean values than *P. hyperborea*—the lateral sepals, labellar blade and labellar spur all approximate 3.5 mm (the Hawaiian *P. holochila* appears to have similarly sized floral organs, though we have been unable to obtain sufficiently accurate dimensions from previous descriptions: Hillebrand, 1888; Kränzlin, 1897–1904). The two smaller-flowered Azorean species plus *P. hyperborea* all have viscidial separation that approximates 0.8 mm, suggesting that further reducing this distance would cause the viscidia to interfere with each other and/or preclude access to the nectariferous spur.

### Invasion of Iceland by *Platanthera hyperborea*

Constructed of basalts upwelling from the Mid-Atlantic ridge that is creating the North American and Eurasian tectonic plates, Iceland has existed above the waves for at least 15 Myr (Kristjansson, Hurdurson & Audunsson, 2003). The island was buried deep under Quaternary ice sheets as little as 15 kyr ago, and most of its land area did not become free of ice until the end of the Younger Dryas period, *ca* 11 kyr ago (Buckland & Dugmore, 1991; Ingolfsson, Norodahl & Schomacker, 2010). It therefore seems likely that the components of the island’s flora have arrived only recently from North America and especially mainland Europe. Although Iceland encompasses an area of 103,000 km^2^, only 23% of the island is currently classified as vegetated rather than barren.

It is not therefore surprising that Iceland supports only *ca* 483 indigenous plus naturalised species of vascular plant (of these, *ca* 435 are angiosperms). A remarkable 97% of these species are shared with Norway but only 66% with Greenland. Despite the fact that westerly winds prevail in Iceland, only eight Icelandic plant species—including *P. hyperborea*—occur in North America but not mainland Europe (cf. Buckland & Dugmore, 1991; Wasowicz et al., 2014; NAT, 2015). This asymmetry in immigration directions is reflected in Iceland’s impoverished orchid flora. The island has accumulated only seven orchid species, all but the rarest (*Neottia ovata*) being specialists in boreal environments: *Pseudorchis straminea*, *Platanthera hyperborea*, *Neottia cordata*, *Corallorhiza trifida*, *Dactylorhiza viridis* and *D. maculata islandica* (Kristinsson, 2010). All of these species except *P. hyperborea* also occur widely in North Norway, a region that presently supports 26 orchid species (Strann & Bjerke, 2010). In contrast, only the first four boreal species are shared with Greenland, where they are accompanied by only one orchid that has not yet reached Iceland, namely *Galearis rotundifolia* (Campbell, 2010). Moreover, *P. hyperborea* and *Pseudorchis straminea* are the only native orchids of Greenland that have succeeded in migrating to its east coast—around Scoresby Sound in the case of *P. hyperborea*, a mere 360 km from Iceland’s northwestern promontory, Westfjords (it is a far more challenging 1,200 km journey to Reykjavik from the more extensively vegetated coastline near the southern tip of Greenland). Thus, it is possible that *P. hyperborea s.s.* is the only orchid species to have reached Iceland from North America rather than from northern Europe.

This observation raises the question of whether *P. hyperborea* has any properties that would assist its eastward colonisation of Iceland. The great majority of north-temperate orchids lack intrinsic sterility barriers and thus conform to Baker’s Law; the ability to undergo uniparental reproduction is likely to be advantageous in colonising individuals that, by definition, will have little or no access to potential reproductive partners upon arrival (cf. Baker, 1955; Roberts & Bateman, 2006; Cheptou, 2012). Hence, a single dust-seed of an orchid is in theory capable of establishing a new population on an oceanic island, provided that through good fortune it encounters a suitable mycorrhizal partner (Bateman & Devey, 2006; Bateman et al., 2014).

We can gain some hints regarding the mobility of *P. hyperborea* seed from long-term studies of the small volcanic island of Surtsey (Fig. 1), which emerged about the sea in 1965, approximately 18 km from the also comparatively recently formed island of Heimaey (which lacks records of *P. hyperborea* according to the Global Biodiversity Information Facility)—45 km offshore, and only a little more distant from our Skogar study site. Careful monitoring of the dates of arrival of botanical immigrants gave 2003 as the first year of flowering of *P. hyperborea* on Surtsey (Magnusson, Magnusson & Fridriksson, 2009), indicating arrival there in *ca* 2000—35 years after the sterile landscape first became available for colonisation. This observation raises the question of whether invasion of Surtsey by a suitable mycorrhizal partner was an essential pre-requisite to successful colonisation by the orchid. Unfortunately, we are not aware of any study of the mycorrhizae associated with *P. hyperborea s.s.* Currah, Smreciu & Hambleton (1990) reported the presence in plants of *“P. hyperborea”* (presumably actually *P. aquilonis*) from Alberta, Canada, of mycorrhizae that proved to be members of basidiomycete families that are widely known to form generalist partnerships with temperate orchid species: Ceratobasidiaceae (*Moniliopsis*) and Tulasnellaceae (*Epulorhiza*). When considered together with its widespread distribution and broad habitat preference, these limited data suggest that *P. hyperborea* is a mycorrhizal generalist and that fungal partners are unlikely to impose a serious constraint on its migration.

As described by Bateman, Rudall & Moura (2013, p. 45), “long-distance airborne dispersal is, by definition, likely to entail both an intense genetic bottleneck and a strong founder effect through the immigrant being in at least some ways genotypically and phenotypically unrepresentative of the source population. And once it has successfully established its first colony on the island, the small founder population, essentially free of a serious risk of further immigration of conspecific seeds, will be especially vulnerable to genetic drift (e.g., Tremblay et al., 2005).” This combination of factors provides an excellent context for the rapid acquisition of facultative autogamy, should the Section *Limnorchis* lineage that reached Iceland not have already acquired this ability while residing in Greenland or possibly Canada. A relatively high percentage of ocean island endemic (and boreo-arctic mainland) species become autogamous, presumably to free them from reliance on what will at best be a limited spectrum of potential pollinators—in other words, to offer reproductive assurance (cf. Cheptou, 2012).

More detailed molecular investigations of populations of *P. hyperborea* and its close relatives in North America and especially Greenland are needed to test the hypothesis that this was the route taken by the lineage in reaching Iceland. The apparent lack of phenotypic discontinuities among the Icelandic populations (Figs. 9 and 10), together with the comparative infrequency of invasions of flowering plant species from North America, suggest that the *Platanthera* lineage invaded Greenland only once. However, the ITS data (Fig. 6) could be taken as suggesting that the ribotype found by us at Soheimajokull represents a more recent invasion of Iceland compared with the slightly more evolutionarily derived ribotype that characterises the remaining populations and appears on current evidence to be unique to Iceland. The enigmatic placement in the ITS tree of the Alaskan sample previously attributed to *P. ‘aquilonis’* discourages more confident interpretation.

### Strong evolutionary parallels between Section *Limnorchis* and Section *Platanthera*

Comparison of the patterns of evolution and migration between Section *Platanthera* and Section *Limnorchis* reveals many parallels. Both groups are molecularly distinct, being subtended by long and robust phylogenetic branches (Bateman et al., 2009; Bateman, Rudall & Moura, 2013). However, within each group, several sets of populations suspected from subtle morphological differences to represent different species overlap in the equally subtle suites of ribotypes that they exhibit (Figs. 5 and 6). Indeed, the ITS tree for the *dilatata-hyperborea* group (Fig. 6) bears a striking resemblance to the ITS tree for the *bifolia-chlorantha* group (Fig. 8 of Bateman et al., 2014); each is characterised by low molecular divergence, imperfect correspondence of ribotypes with morphologically circumscribed taxa, and unique island ribotypes that are comparatively derived. Widespread geographical sampling of all relevant taxa for both detailed morphometric and detailed population genetic analyses are still required in order to confidently identify species boundaries (Bateman, 2001; Bateman, 2012).

Assuming that the majority of the Linnean epithets in current use in these groups do indeed represent *bona fide* species, the closely similar ribotypes and paucity of morphological autapomorphic character states within each group together suggest that speciation occurred comparatively recently—most likely within the last 11,000 years in the case of *P. hyperborea*, should it prove to be confined to Iceland and Greenland. It could in theory be argued that these lineages might be older but subject to ongoing gene-flow; however, hybridisation is rare among the three Azorean species (Bateman et al., 2014; PV Araujo, pers. comm., 2014) and only one species appears to be present on Iceland. Certainly, neither the Icelandic nor the Azorean species are credible as potential relictual lineages. In both island systems, the ITS data are ambiguous regarding whether the pertinent *Platanthera* lineage invaded the islands once or twice.

**Figure 16:**
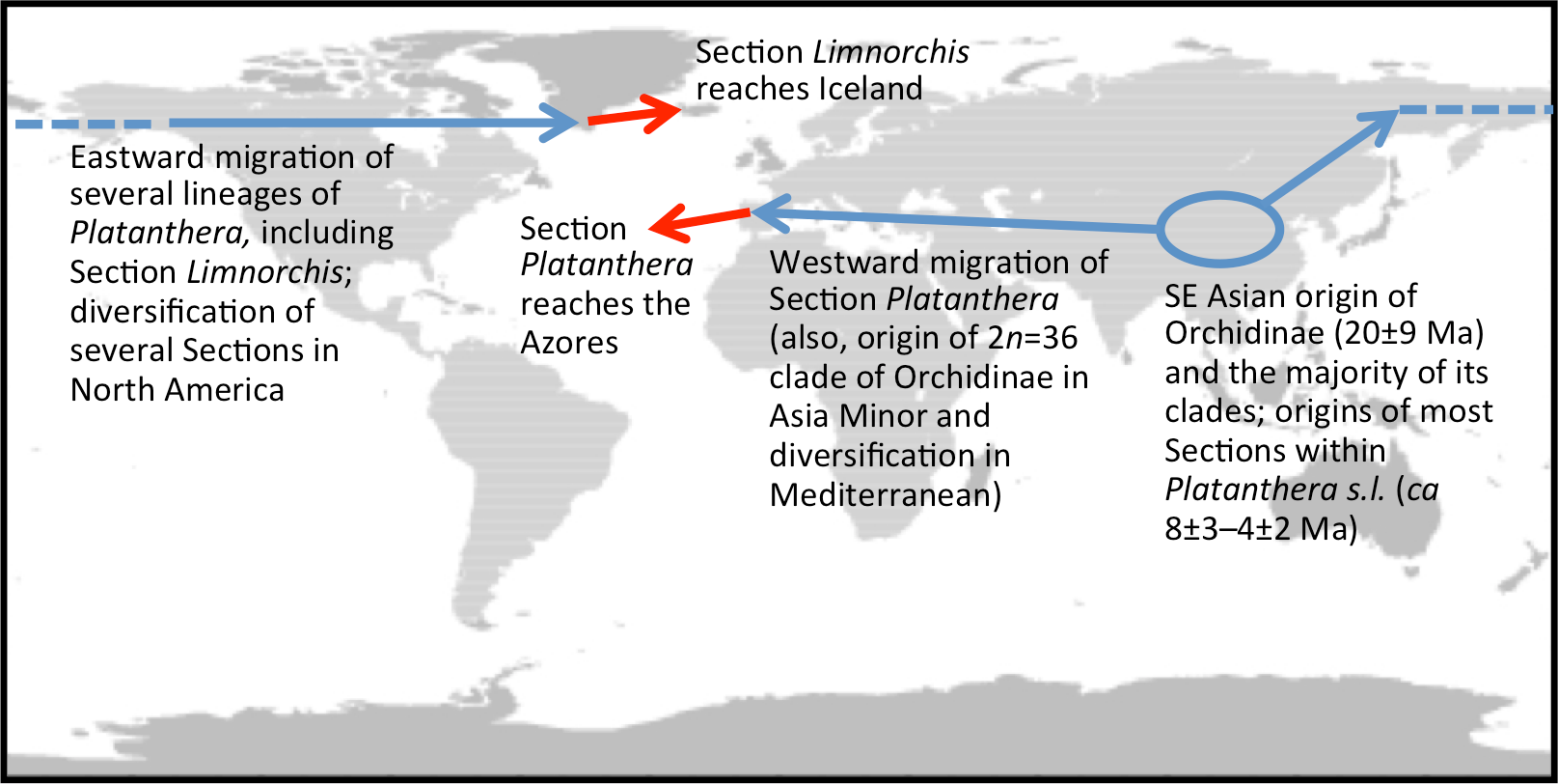
Scenario suggesting how the two most phylogenetically divergent lineages within the genus *Platanthera* ultimately reached adjacent mid-Atlantic archipelagos by migrating (and evolving) circumboreally but in opposite directions.

The evolutionary and migratory journeys undergone by the two lineages also bear comparison. Most molecular phylogenies of subtribe Orchidinae, including the most recent (Tang et al., 2015), strongly suggest that the subtribe originated in southeast Asia at 20 ± 9 Ma (Sramkó et al., 2014). Moreover, most of those topologies also suggest that the genus *Platanthera s.l.*, and most of its eight monophyletic taxonomic Sections (cf. Hapeman & Inoue, 1997; Bateman et al., 2009), also originated in southeast Asia—the genus at 8 ± 3 Ma and the Sections a little later. Most of the Section-level lineages then migrated both eastward and westward, speciating as they migrated (Fig. 16). Based on present evidence, Section *Platanthera* has achieved its greatest species-level diversity in western Europe whereas Section *Limnorchis* has done so in North America, each lineage having expanded almost halfway around the globe but predominantly in opposite directions. The ‘final push’ into the Atlantic Ocean is hypothesised to have carried the Section *Platanthera* lineage *ca* 1,600 km westward from Iberia to the Azores, and the Section *Limnorchis* lineage *ca* 1,200 km eastward from southern Greenland to Iceland (Fig. 16). It is clear that the three Azorean species originated on the archipelago, whereas on present evidence it seems more likely that *P. hyperborea* originated *before* migrating to Iceland.

Speciation on both islands involved substantial reduction in flower size. However, the percentage diminution in size was probably much greater on the Azores, where each of the two most credible ancestors is large-flowered, than that required to generate *P. hyperborea* from its most likely ancestor, the medium-sized-flowered *P. dilatata* (Fig. 6). Bateman et al. (2014) argued that the environmental conditions pertaining on islands increase the probability of anagenetic speciation, and suggested that the majority of the 15 orchid species found on the Macaronesian islands were the result of anagenetic speciation, arguing that only a single case of cladogenesis was required to explain the Azorean *Platanthera*s. We hypothesise that *P. hyperborea* may also have originated through anagenetic speciation. In terms of phenotypic change, Bateman et al. (2014) argued for a speciation process that involved paedomorphosis, the size reductions also leading to shape changes in the case of at least some floral organs.

However, the present analysis (Figs. 11 and 12) suggests that, at the level of putative species, most metric characters operate within strong developmental and/or functional constraints that deter radical deviations in the shapes of floral organs, instead favouring broadly allometric transitions that alter size but not shape. These constraints may have aided the phenotypic convergence in many floral characters between *P. hyperborea*, the two smaller-flowered Azorean species and possibly also the Hawaiian *P. holochila*—convergence that generated broadly similar sizes of most floral organs. In these species, the gynostemium at least appears to have been reduced as radically as is feasible without becoming severely dysfunctional. Only the dominant mode of pollination seems to have diverged greatly between the Azorean species, which we believe still rely primarily on allogamy through micro-moth pollinators, and *P. hyperborea*, where premature desiccation of the gynostemium has generated a remarkably sophisticated and well-adapted mode of cleistogamous autogamy.

## Additional Information and Declarations

### DNA Deposition

The following information was supplied regarding the deposition of DNA sequences: GenBank: KR074429 and KR074430.
